# Copying fidelity of functional and non-functional features in ni-Vanuatu children: A transmission chain study

**DOI:** 10.1371/journal.pone.0274061

**Published:** 2023-02-09

**Authors:** Anne Sibilsky, Heidi Colleran, Dominik Deffner, Daniel B. M. Haun

**Affiliations:** 1 Department of Comparative Cultural Psychology, Max Planck Institute for Evolutionary Anthropology, Leipzig, Germany; 2 Leipzig Research Center for Early Child Development, Leipzig University, Leipzig, Germany; 3 *BirthRites* Independent Max Planck Research Group, Max Planck Institute for Evolutionary Anthropology, Leipzig, Germany; 4 Department of Human Behavior, Ecology and Culture, Max Planck Institute for Evolutionary Anthropology, Leipzig, Germany; 5 Science of Intelligence Excellence Cluster, Technical University Berlin, Berlin, Germany; 6 Center for Adaptive Rationality, Max Planck Institute for Human Development, Berlin, Germany; University of Queensland, AUSTRALIA

## Abstract

Observational learning plays a key role in cultural transmission. Previous transmission chain experiments have shown that children are able to maintain information across multiple generations through observational learning. It still remains unclear how the transmission of functional vs. non-functional information and the effect of being observed unfold across age in different communities. Here, we examine children’s copying fidelity in observational learning of 5- to 13-year-olds from five different communities in Vanuatu, both individually (n = 263, 144 boys) and throughout a transmission chain of five to six children (n = 324, 178 boys). We additionally varied the functionality of the feature being copied (shape vs. color) and the copying context (observed vs. unobserved). Further, we also study developmental and cultural variation in the interaction of features and conditions. We find that children transmit the functional feature *shape* more faithfully than the non-functional feature *color*, both in the dyadic transitions as well as the transmission chains with an increasing tendency to do so as they get older. The age patterns show greater variation between communities for color than for shape. Overall, we find that being observed shows no uniform effects but influences transmission differently across communities. Our study shows that children are prone to passing on a functional feature across multiple generations of peers. Children copy non-functional features as well, but with lower fidelity. In sum, our results show children’s high propensity and developing abilities for observational learning, ultimately allowing for effective cultural transmission.

## Introduction

Culture can be defined as a set of behavioral traditions that are transmitted by social learning [1, 2]. Observational learning, one important social learning mechanism, has been ascribed a key role in human cultural transmission [3]. The observational learning of children is of special interest: it equips children with the essential skills they need to become fully-fledged members of the society they live in [4, 5]. As such, children have been labelled as “cultural magnets” [3], and childhood is regarded as a period of massive cultural assimilation [6]. Here, we study observational learning across middle childhood in a country with only approximately 272,000 inhabitants but 138 indigenous languages. Assuming language groups approximate cultural groups, Vanuatu’s linguistic diversity indicates extensive cultural diversity. We thereby aim to expand the understanding of both childhood learning and human cultural diversity.

Observational learning is “learning by watching the behavior of others” [3]. Previous research has shown that children are able to learn even if they cannot see the actual action but only its result through so-called *end-state copying*: 19-month-old British infants are able to emulate a required object transformation when exposed to the initial and end-states of an object [7] and four- to five-year-old British children can build a taller spaghetti tower after observing a tower built by others [8].

Combining multiple successive events of end-state copying generates a *transmission chain* [9, 10] which allows researchers to simulate the transmission of cultural information across generations (i.e., cultural evolution) under controlled laboratory conditions: participants in each position of the chain are presented with socially generated information of participants in earlier positions of the chain. Thereby, the fidelity of transfer over repeated transmission events can be tracked by rating the similarity of the respective results. Reindl and Tennie [11] asked four- to six-year-old British and German children to build a spaghetti tower as tall as possible while seeing the towers built by the two previous children. While children’s towers showed no indication for cumulative culture (i.e., higher towers in later positions), the similarity of towers was higher within than between chains “indicating the presence of chain-specific design traditions” [11]. In addition, Flynn and Whiten [3] found high fidelity in *how* children achieve an end-state in chains of five children each: 3- and 5-year-olds were asked to remove polystyrene beads from a transparent box using one of two techniques. Children mostly followed the approach they witnessed and thereby were more successful than children in a control condition.

In the present study, we investigate children’s fidelity to copy end-states in a transmission chain across middle childhood. We asked children to build a marble run from various building blocks and displayed the marble run built by the previous child. To build a marble run, children could use building blocks in different shapes and colors. The functionality of these two features varies: While the degrees of freedom of the shape feature were restricted, given the aim of building a marble run, the color of the bricks had no influence on the functionality. Therefore, we consider shape as a *functional* feature and color as a *non-functional* feature.

In the following we explain and state our hypotheses H1 to H7 (see also Table 3). We hypothesize that shape will be transmitted more faithfully than color due to the varying functionality of the features [H1]. The reason we predict that color features would be transmitted at all is based on prior studies, relating the copying of non-functional features to chidlren’s other, non-informational motivations. Copying a non-functional feature might for example be motivated by social reasons (e.g., affiliation, secure group benefits). To test the possible relevance of social motivations in children’s copying, we varied the social context of the situation and tested children in an observed and an unobserved condition. We assume that children copy *shape* (functional feature) equally often in an observed and an unobserved condition [H2a] but copy *color* (non-functional feature) more often when they are observed [H2b]. Previously, in a study on conformity [12]), we showed higher sensitivity to social influences among ni-Vanuatu girls than boys. Therefore, we expect a higher observation effect in girls than boys also in the present study [H3].

To better understand the development of copying fidelity and its cultural variability, we further assessed developmental and cultural variation in the interaction of features and conditions: we expect that older children copy shape [H4a] and color [H4b] more faithfully due to their overall increased cognitive abilities. The age effect might further interact with condition: Previous research has shown that specifically the tendency to copy causally unnecessary actions (so-called “over-imitation” [13, 14]) tended to be higher in older than younger children among 3- to 9-year-old Ovambo and Hai||om children [15] and 3- to 11-year-old U.S. American children [16] (but remained stable in German children [15] and 4- to 11-year-old British children [17]). Furthermore, 3- to 9-year-old Ovambo and Hai||om children [15] also show an increased observation effect with age, leading to higher rates of copying non-functional acts under observation with age. Consequently, we expect an increased observation effect in older children in our study [H5].

Psychologists also increasingly recognize the need for more diverse samples that capture the breadth of human conditions and increase the generalizability of findings across populations [18, 19]. To study cultural variation, we conducted our study in five communities belonging to different ethnolinguistic groups of one island in the Republic of Vanuatu, an archipelago in the southwestern Pacific. Studying copying fidelity across communities in a culturally diverse society allows us to examine cultural variation while minimizing the influence of factors such as varied ecology or living conditions that normally affect cross-country comparisons. As we assume non-functional features to be more culturally variable than functional features, we expect more variation across communities in copying color than in copying shape, both in terms of the general extent of copying [H6a] and also its age patterns [H6b]. Previously, we showed that the availability of a child’s behavior to peers can trigger more conformist responses [12]. Therefore, we expect that being observed might have the aggregate effect of exaggerating potential between-community differences in the extent [H7a] and age patterns [H7b] of copying.

Lastly, in addition to the analysis of individual-level copying, we also investigated copying fidelity throughout the transmission chain. This allowed us to see how the hypothesized influence of feature functionality and copying context on individual-level copying would scale up and accumulate over multiple generations.

The cultural diversity in Vanuatu not only allows us to study baseline cultural differences in the end-state copying of children, it could also be associated with an exaggerated importance of high fidelity copying. In addition to three official languages (Bislama, French and English), around 138 indigenous languages of the Oceanic subgroup of the Austronesian language family are spoken in Vanuatu [20] by a population of approximately 300,000 inhabitants [21]. According to Francois [22], this linguistic diversity emerged because language fulfills an emblematic function: it anchors communities in social and geographic space. A necessary condition for the rapid diversification in languages that occurred since first occupation about 3,000 years ago [23, 24] could be high fidelity copying as a way to faithfully transmit linguistic and cultural information to the next generation. This again makes Vanuatu particularly well suited for the study of high fidelity copying.

## Methods

### Ethics

The study protocols and their application in Vanuatu were approved as part of a broader project using non-invasive behavioral experiments and focusing on early child development, carried out at the Leipzig Research Center for Early Child Development in Germany, and reviewed by the Ethics Committee of the Leipzig Research Center for Early Child Development in Germany (reference number 169/17-ek). In Vanuatu, permission to carry out the research was given by the Cultural Council of the Vanuatu Cultural Center which regulates all research in the country.

In all participating communities, we obtained informed written consent from the principal of the school in which the experiments were carried out, following extensive discussion of the study protocols. We additionally obtained written consent from the parents of participating children for the collection of experimental data (i.e., study participation, anonymous video recording, and the further usage of the data for scientific purposes) from their children.

### Field site

We conducted our study on the principal island of Efate, which has a population of approximately 92,000 inhabitants [21]. Efate belongs to Shefa province and is also home to Vanuatu’s capital Port Vila. There are at least three indigenous languages on Efate, Ifira-Mele, Nakanamanga and Nafsan [25], but many other indigenous languages of Vanuatu are now spoken in a wide variety of communities due to in-migration from all over the country (Sibilsky A. A Theme and its Variation: Majority-biased Learning in ni-Vanuatu Children. Unpublished PhD. Leipzig: Leipzig University; 2021. [Unpublished]).

Data were collected in five different schools in distinct communities. According to the residents, these communities each speak a different language. Three of them are rural communities with several hundred residents, located between 25 to 55 km from the capital and accessible in 1 to 1.5 hours by public transportation. The other two study sites have a few thousand inhabitants and are located 7 to 10 km from the capital, which can be reached in 20 to 30 minutes by public transportation. Most of our participants live in brick houses (61%), corrugated-iron huts (22%) or houses made of local materials (15%) (data from interviews with 113 parents of participating children, from (Sibilsky A. A Theme and its Variation: Majority-biased Learning in ni-Vanuatu Children. Unpublished PhD. Leipzig: Leipzig University; 2021. [Unpublished]). Almost all have electricity (96%) either through solar energy (72%) or power lines (27%). Most adults own smartphones (73%), and some have computers (29%) or TVs (22%). Most people have formal education up to primary (43%) or secondary school (50%), but not beyond. About one-third of people are employed (22%) or self-employed (10%). Almost all people have a garden (89%) and use it for their subsistence (95%). The majority of ni-Vanuatu practice slash-and-burn horticulture, and grow staple crops such as yam, taro, banana and manioc (cassava), as well as a variety of vegetables. In addition, they regularly fish (65%), hunt (20%), raise livestock (54%) and buy market-based products. Nearly half of the people reported experiencing economic hardship in the past year (42%) (for more information on all communities, see (Sibilsky A. A Theme and its Variation: Majority-biased Learning in ni-Vanuatu Children. Unpublished PhD. Leipzig: Leipzig University; 2021. [Unpublished]) and Supplementary Methods 1 in [26]).

### Sample recruitment and selection

Data were collected between July and September 2018. Participants were recruited out of children from class 1 to 4 who had parental consent. Priority was given to children for whom data already existed, either from our previous study on conformity in 2017 [12] and/or interviews with parents of participating children (Sibilsky A. A Theme and its Variation: Majority-biased Learning in ni-Vanuatu Children. Unpublished PhD. Leipzig: Leipzig University; 2021. [Unpublished]). Usually, we gave consent forms to all children of classes 1 to 4 and conducted the study with almost all for whom we had approval. There were no other criteria, selection was opportunistic. Where possible, same-sex children were recruited out of the same classroom.

### Design and study setup

Instructions to the children were standardized and given in Bislama, a *lingua franca* that is understood by all participating individuals (see S1 Section in S1 File for a more detailed version of the procedure). Two trained experimenters were present. Experimenter one was a local woman not from any of the participating communities, who gave the instructions to the child. Experimenter two was either a German master’s student or the first author (both White women), who took care of the video recording, counter balancing, live coding and procedure alignment.

**Fig 1:**
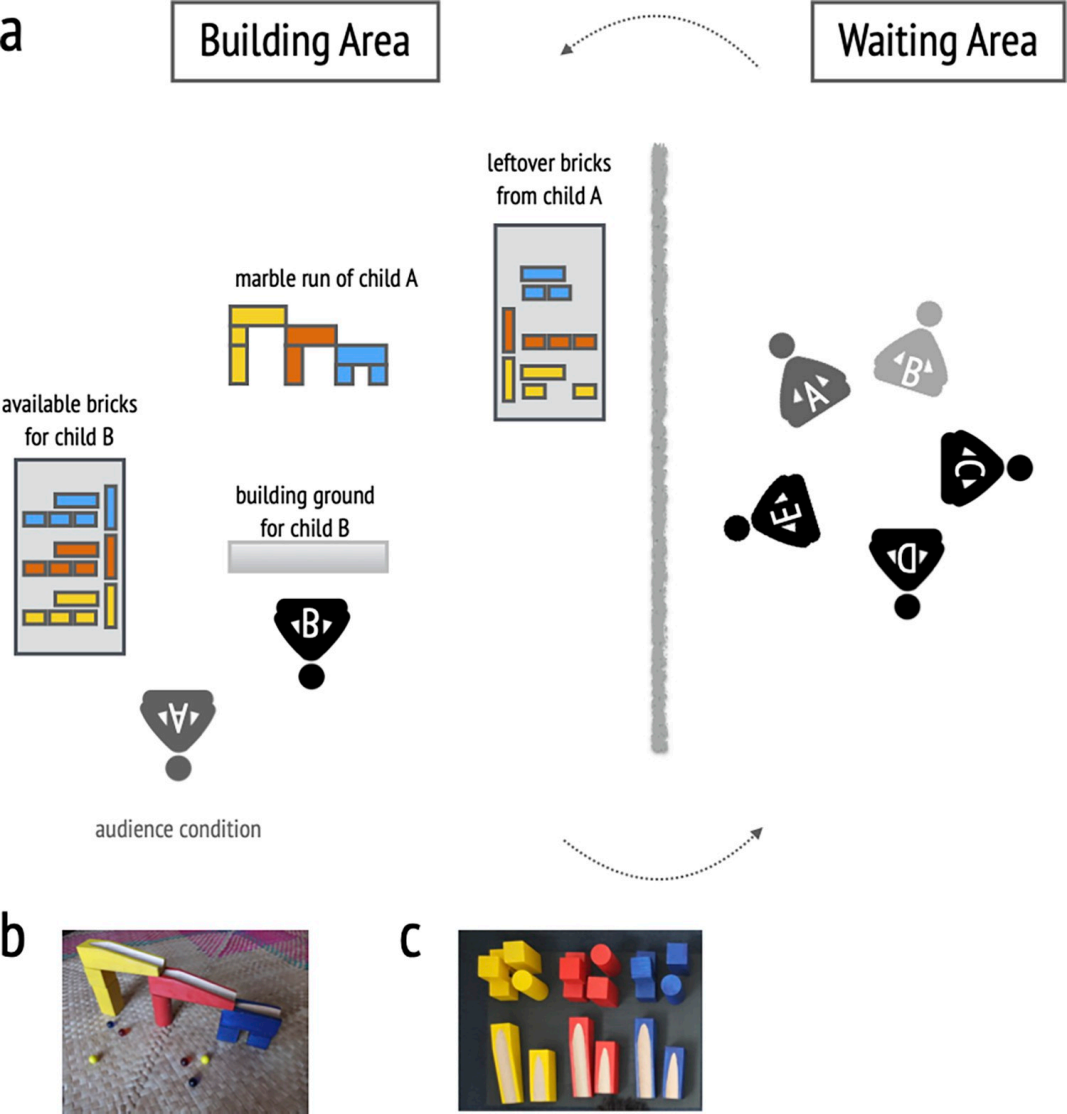
Study design and setup. Note. a) is a schematic illustration of the study setup with the building area on the left and the waiting area on the right which are separated by a curtain. Depending on the condition child A is in sitting in the building area (observed condition) or in the waiting area (unobserved condition) while child B is tested. b) shows the initial marble run given by the experimenter. Panel c) shows the set of building blocks provided to the child.

Experimenter two called five (unobserved condition) to six (observed condition) children into the study room, telling them that we would like to play a game. The study room was divided into a “waiting area” and a “building area”, separated by a waist-high curtain (Fig 1A and 1B). The waiting area was used for warm-ups and relaxation for children not currently building a marble run, while the building area was used to assess children’s copying fidelity, keeping them undisturbed but in close proximity to the other children.

In both conditions (observed and unobserved), the procedure was as follows: The children were welcomed by the two experimenters, who introduced themselves and asked them to sit in a circle in the waiting area. In the warm-up phase children were asked to sort a scattered pile of building blocks to familiarize them with the study materials. Once finished sorting, children drew lots to determine the order of entering the building area. The first selected child followed experimenter one into the building area, while the other children were given pencils and paper for drawing, supervised by experimenter two.

The child sat down in front of a marble run. For the first child in every group, the experimenter showed them a pre-prepared marble run (Fig 1C), all other children were shown the marble run from the previous round. The experimenter then demonstrated the function of the marble run by rolling down one or two marbles. This step was implemented to ensure a certain kind of building by specifying its use. Finally, the experimenter presented the child with a set of building blocks and asked them to build their own marble run. We framed the instruction in a way that children neither felt pressured to exactly copy the marble run nor to build a completely different marble run. To ensure some degree of comparability, we stuck an adhesive strip on the floor to indicate children to build a linear marble run.

Experimenter one then went to the waiting area until the child rang a bell or a maximum of four minutes had passed. She then returned to the construction area and asked the child to show their marble run. The child subsequently returned to the waiting area while experimenter one took a picture of the marble run and prepared the building area for the next child. To do this, she either dismantled the marble run of the child’s predecessor (leaving the just-built marble run as a model for the next child) or dismantled the child’s marble run if it consisted of fewer than five (*n* = 4) or more than eleven (*n* = 6) bricks to avoid very simple and very complex marble runs (leaving the previously built marble run as a model for the next child but pretending that the current child had built it), and prepared the building blocks for the next child. We dismantled very simple and very complex marble runs to keep the difficulty of each model run stable within a certain range, thus measuring children’s propensity to copy rather than their ability to do so. Then, experimenter one called the next child from the waiting area and the process was repeated.

In the observed condition, starting with the second child, participants were not asked to return to the waiting area after building a marble run, but stayed in the building area to observe the following participant by sitting diagonally behind the current participant. Experimenter one emphasized that the observing child should do so quietly, without interacting. Once the last child built their marble run, we thanked children and they went back to their classrooms.

### Participants

80 groups took part in the experiment with a total of 394 children (observed condition: 39 groups, 203 children; unobserved condition: 41 groups, 191 children). Data from 60 children (15% of all children) had to be excluded because they did not build anything but instead looked around or played with the marble (*n* = 44), they dismantled the marble run that they built themselves or the previous marble run (*n* = 6), they built something without orientation on the adhesive strip or without a horizontal ramp (*n* = 6), they used less than five bricks (*n* = 2), they accidently participated twice (*n* = 1), or because there was an experimenter error with camera (*n* = 1).

For the analysis of individual copying another 71 children were excluded from the remaining 334 children, because they were in the first position (*age range (years)*: 6.42–13.25; *M* = 9.12; *SD* = 1.51; 37 boys) and thus their data could not be regarded as “copying peers”. The final sample includes 263 children (*age range (years)*: 5.92–13.08; *M* = 9.12; *SD* = 1.44; 144 boys) with 136 children in the observed condition and 127 children in the unobserved condition (see Table 1 and S1 Fig in S1 File for the community-wise age distribution).

**Table 1:** Sample composition for the analysis of individual copying fidelity: Age distribution of children by community and condition for the total sample.

Community		N (male)	mean age	sd age	range age
A		42 (24)	8.9	1.2	6.17,11.00
	observed	22 (15)	9.4	1.1	7.50; 11.00
	unobserved	20 (9)	8.2	1.1	6.17; 10.00
B		63 (32)	9.2	1.4	5.92,12.00
	observed	28 (15)	9.1	1.4	5.92; 10.92
	unobserved	35 (17)	9.3	1.5	6.67; 12.00
C		74 (38)	9.3	1.5	6.33,13.08
	observed	37 (18)	9.4	1.6	6.33; 13.08
	unobserved	37 (20)	9.2	1.4	6.58; 13.08
D		29 (14)	8.8	1.4	6.00,11.50
	observed	16 (7)	9.0	1.2	6.58; 10.67
	unobserved	13 (7)	8.4	1.6	6.00; 11.50
E		55 (36)	9.1	1.5	6.00,12.25
	observed	33 (24)	8.9	1.7	6.00; 12.25
	unobserved	22 (12)	9.3	1.2	6.92; 11.17

For the analysis of copying in the transmission chain another 10 children were excluded from the remaining 334 children. These children were successors of children whose marble run had to be dismantled. However, we then accidentally provided the experimenter marble run instead of the marble run of the predecessor. We consider this to be an intervention in the chain of transmission compared to how it would have proceeded uninfluenced and therefore excluded all of the following children. The final sample includes 324 children (*age range (years)*: 5.92–13.25; *M* = 9.12; *SD* = 1.46; 178 boys) with 173 children in the observed condition and 151 children in the unobserved condition (see Table 2 and S2 Fig in S1 File for the community-wise age distribution).

**Table 2:** Sample composition for the transmission chain analysis: Age distribution of children by community and condition for the total sample.

Community		N (male)	mean age	sd age	range age
A		53 (31)	8.9	1.4	6.17,13.25
	observed	28 (19)	9.4	1.3	7.42; 13.25
	unobserved	25 (12)	8.4	1.3	6.17; 11.08
B		80 (41)	9.2	1.4	5.92,12.00
	observed	35 (19)	9.2	1.3	5.92; 10.92
	unobserved	45 (22)	9.3	1.4	6.67; 12.00
C		91 (46)	9.3	1.4	6.33,13.08
	observed	48 (22)	9.4	1.6	6.33; 12.92
	unobserved	43 (24)	9.2	1.3	6.58; 13.08
D		33 (16)	8.8	1.5	6.00,12.00
	observed	20 (9)	9.2	1.3	6.58; 12.00
	unobserved	13 (7)	8.3	1.6	6.00; 11.50
E		67 (44)	9.1	1.6	6.00,12.5
	observed	42 (30)	9.0	1.7	6.00; 12.50
	unobserved	25 (14)	9.2	1.5	6.92; 11.83

### Coding and reliability

We coded the data differently for the analysis of individual copying fidelity and the transmission chain analysis. For individual copying fidelity, the study was coded by experimenter two who rated the copying fidelity of children based on the pictures of the marble runs. The marble runs were divided into coding units, with ramps and cubes being one unit and all other blocks being two units (because two cubes could replace them, see S3 Fig in S1 File for a coding example). These units were then counted in terms of a) the maximum possible number of matching units (i.e., depending on which marble run had more units, the number of units of the previous marble run or the child’s marble run), b) the number of matching shape units between the marble run of the child and the previous marble run, and c) the number of matching color units between the marble run of the child and the previous marble run.

85 cases (25% of 334 children) were re-coded by a trained coder blind to the hypotheses. Interrater reliability, calculated with R and the package ‘irr’ [27], showed substantial agreement (*Cohen’s kappa*_*unit maximum*_ = 1, *Cohen’s kappa*_*matching shape units*_ = 0.729, *Cohen’s kappa*_*matching color units*_ = 0.814, [28]). In cases of disagreement (*n*_*unit maximum*_ = 0 out of 85, *n*_*shape matches*_ = 15 out of 85, *n*_*color matches*_ = 13 out of 85), the rating by experimenter two was preferred due to her greater experience with the coding scheme.

For the transmission of information throughout the chain, we binarised whether the shape and the color of each of the eight building blocks that made up the initial marble run given by the experimenters, which was the same in each group, was reproduced by a child (yes = 1/ no = 0). Once a feature was lost, it could not be rated again in that group: for example, if child A used a red big building block instead of the original yellow big building block, and then child B switched back to a yellow building block, this would still not count as a transmission event.

81 cases (25% of 323 children) were re-coded by a trained coder blind to the hypotheses. Interrater reliability, calculated with R and the package ‘irr’ [27], showed substantial agreement (*Cohen’s kappa = 0*.*94*, [28]). In cases of disagreement (37 out of 1296), the first author checked the pictures again and decided due to her greater experience with the coding scheme.

### Statistical methods

We analyzed all data within a Bayesian modeling framework using the package “rethinking” [29] and “rstan” [30] in R-Studio, R version 4.0.3 [31]. Age was mean-centered.

### Individual copying fidelity

For individual copying fidelity, we wanted to know the probability that an individual matches the shape and color features of the previous marble run. We modeled children’s copying fidelity using a binomial regression model with a logit link function, separately for shape and color features:

y∼Binomial(n,p),

where copying fidelity *y* is the number of matching (shape or color) units, *n* is the maximum possible number of matching units and *p* is the probability that a child copies a given unit. To investigate which features influence this copying probability, *p* was defined as a function of the experimental condition (observed and unobserved), the position of the child in the chain, the community children live in, the child’s age, sex, as well as the potential interactions between sex and condition. We also examined the age pattern of copying in the two conditions across communities by evaluating all 2- and 3-way interactions between age, condition, and community. We used a multilevel model to account for the clustered structure of the data, i.e., multiple children belong to the same experimental group and multiple experimental groups live in the same community, and included varying (or “random”) effects for each child, group and community (see OSF repository for full model code, [32]). All reported values are based on this model.

Results revealed an interaction between condition, community, and age. To interpret this interaction and better understand varying age trends across communities and conditions, we set up a second model and plotted the model predictions. As we were only interested in this interaction, we did not include other variables (i.e., position and sex). We again used a Bayesian multilevel model to account for the nested structure of the data (children within groups, groups within communities) allowing both the slopes of age and condition as well as the slope of the age-condition interaction to vary by community. This accounts for the fact that different community samples include data on different age ranges.

### Transmission chain analysis

In addition to dyadic copying fidelity, we also modeled information flow along the whole transmission chains. For this analysis, we considered for how many iterations of the experiment the building blocks of the original marble run were transmitted, so we ignored the blocks that were introduced at later stages. To compute how far features are expected to travel along the chain, we estimated the transition probabilities for an original feature to be transmitted from the first chain position (i.e., the first iteration of the experiment) to the second, from the second to the third, and so on. This means, conditional on the feature being transmitted up to a certain point, what is the probability that it is also transmitted to the next stage? The outcome variable was whether a given original building block was copied or not by a participant (coded as 1 and 0). We used a Bernoulli likelihood and modeled the six transition probabilities (corresponding to the maximum chain length of six iterations) as ordered-categorical or “monotonic” effects. This means the model assumes that transmission probabilities change in the same direction as we travel along the chain, but the size of the change between, for instance, the first and second stage might differ from the change between the second and third stage. We modeled transmission probabilities separately for the five communities, experimental conditions (observed vs. unobserved) and feature types (shape vs. color) and included individual id and position in the chain as varying effects. The actual transmission stage, which we modeled using monotonic effects, could differ from the position of a child in the chain, because not all children built a marble run that served as a model for the next child in the chain (see *Design and study setup*).

For all analyses, we estimated the posterior probability of parameters conditional on the data using the Hamiltonian Monte Carlo engine *Stan* implemented in Rstan [30] with weakly informative priors to ensure model fit. Inferences are based on 2,000 samples from four chains (after 500 adaptation steps) for a total of 4,000 samples and between 246 to 13462 (individual copying fidelity) and 404 to 6211 (transmission chain) effective samples from the posterior distribution. Model results are reported in detail in S1 Table in S1 File as posterior distributions with mean (β) and 89% percentile interval (PI) on the outcome scale. There we also report the 89% highest posterior density interval (HPDI) of contrasts that we calculated to get the distribution of the expected difference between two levels of a predictor. All analysis scripts and data files are available on the Open Science Framework (https://osf.io/btkgn/?view_only=8722535221b8485d971b3c621c02011a, [32]).

## Results

### Individual copying fidelity

**Fig 2:**
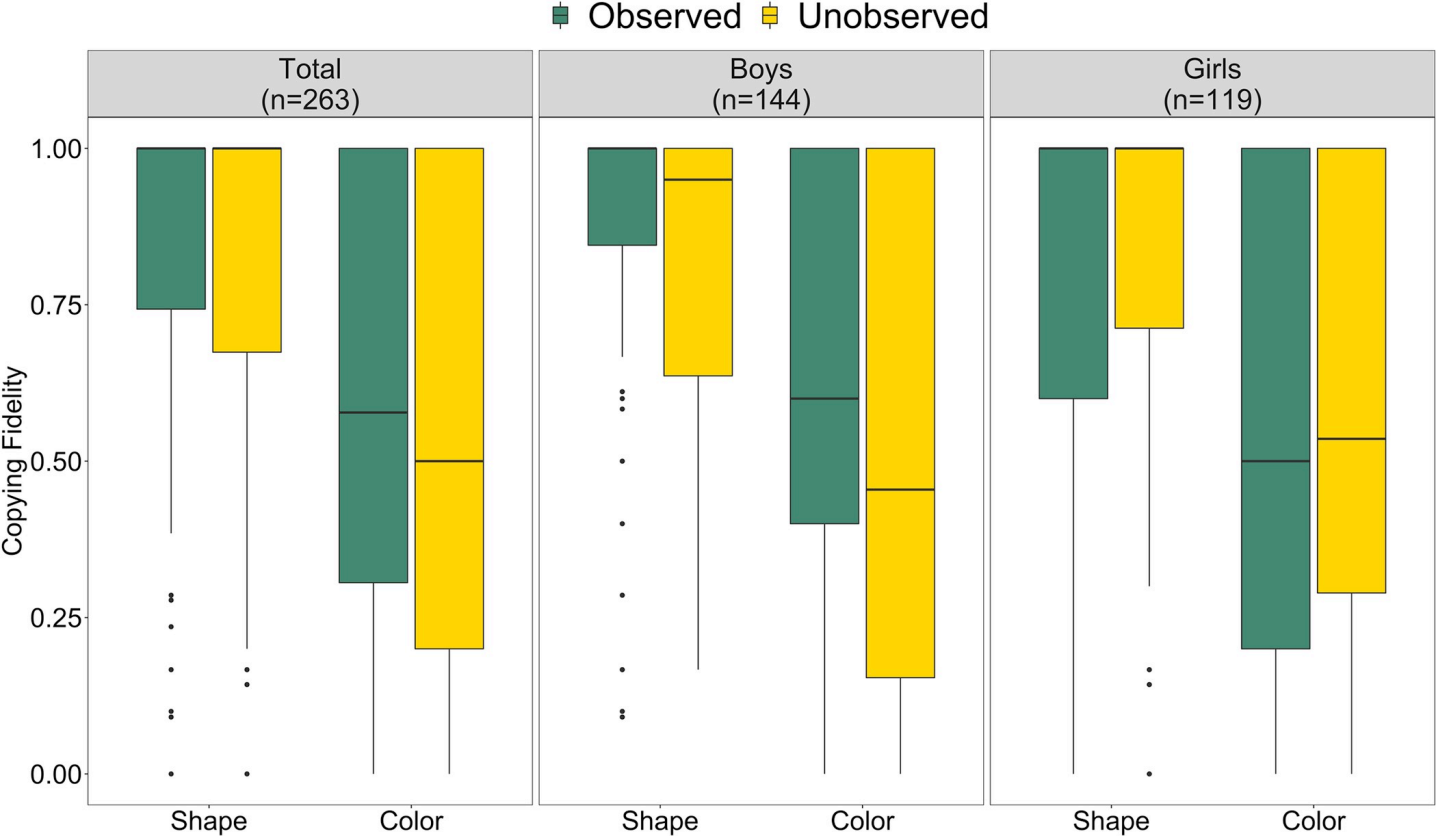
Individual copying fidelity for the total sample, and for boys and girls separately. Note. Individual copying fidelity of the shape and color feature in the observed (green) and unobserved (yellow) condition. a) shows that on average children copy shape more faithfully than color. The boxes represent the interquartile range (IQR), the bold, horizontal lines within the boxes are medians, the upper vertical lines attached to the boxes extend from the hinge to the largest value no further than 1.5 * IQR from the hinge, the lower vertical lines attached to the boxes extend from the hinge to the smallest value at most 1.5 * IQR of the hinge. b) shows that boys tend to copy with slightly higher fidelity when they are observed whereas girls tend to copy with slightly higher fidelity when they are unobserved.

Fig 2 shows individual-level copying fidelity. We find that children copy shape more faithfully than color (*M*_*Shape*_ *= 0*.84; *M*_*Color*_ *= 0*.*57*; 89% HPDI for contrast [0.23, 0.27]; Fig 2A), which is true for both boys and girls (Fig 2B, see S1 Table in S1 File for all values) [H1, see Table 3 for a summary of all hypotheses]. On average, we do not find that children’s copying fidelity (for either shape 89% HPDI [-0.44, 0.79] or color 89% HPDI [-0.44, 0.76]) differed between the observed and unobserved conditions (Fig 2A) [H2a, H2b]. However, boys tended to copy both color (contrast to girls: 89% HPDI [-1.22, 0.35]) and shape (contrast to girls: 89% HPDI [-1.32, 0.26]) features with higher fidelity in the observed compared to the unobserved condition whereas girls show the opposite tendency (Fig 2B) [H3]. Note that distributions for contrasts overlap 0, so our results point to but do not provide conclusive evidence for the observed gender difference.

**Table 3:** Hypotheses and their results.

Hypotheses		Result
H1	Shape will be transmitted more faithfully than color.	Confirmed
H2a	Children copy *shape* equally often in an observed and an unobserved condition.	Confirmed
H2b	Children copy *color* more often when they are observed.	Not confirmed
H3	We expect a higher observation effect in girls than boys.	Not confirmed
H4a	Older children copy shape more faithfully than younger children.	Confirmed
H4b	Older children copy color more faithfully than younger children.	Not confirmed
H5	Older children show compared to younger children an increased observation effect.	Not confirmed
H6a	There is more variation across communities in copying color than in copying shape in the general extent of copying.	Not confirmed
H6b	There is more variation across communities in copying color than in copying shape in its age patterns.	Confirmed
H7a	Being observed exaggerates potential between-community differences in the extent of copying.	Not confirmed
H7b	Being observed exaggerates potential between-community differences in the age patterns of copying.	Confirmed for color, not confirmed for shape

**Fig 3:**
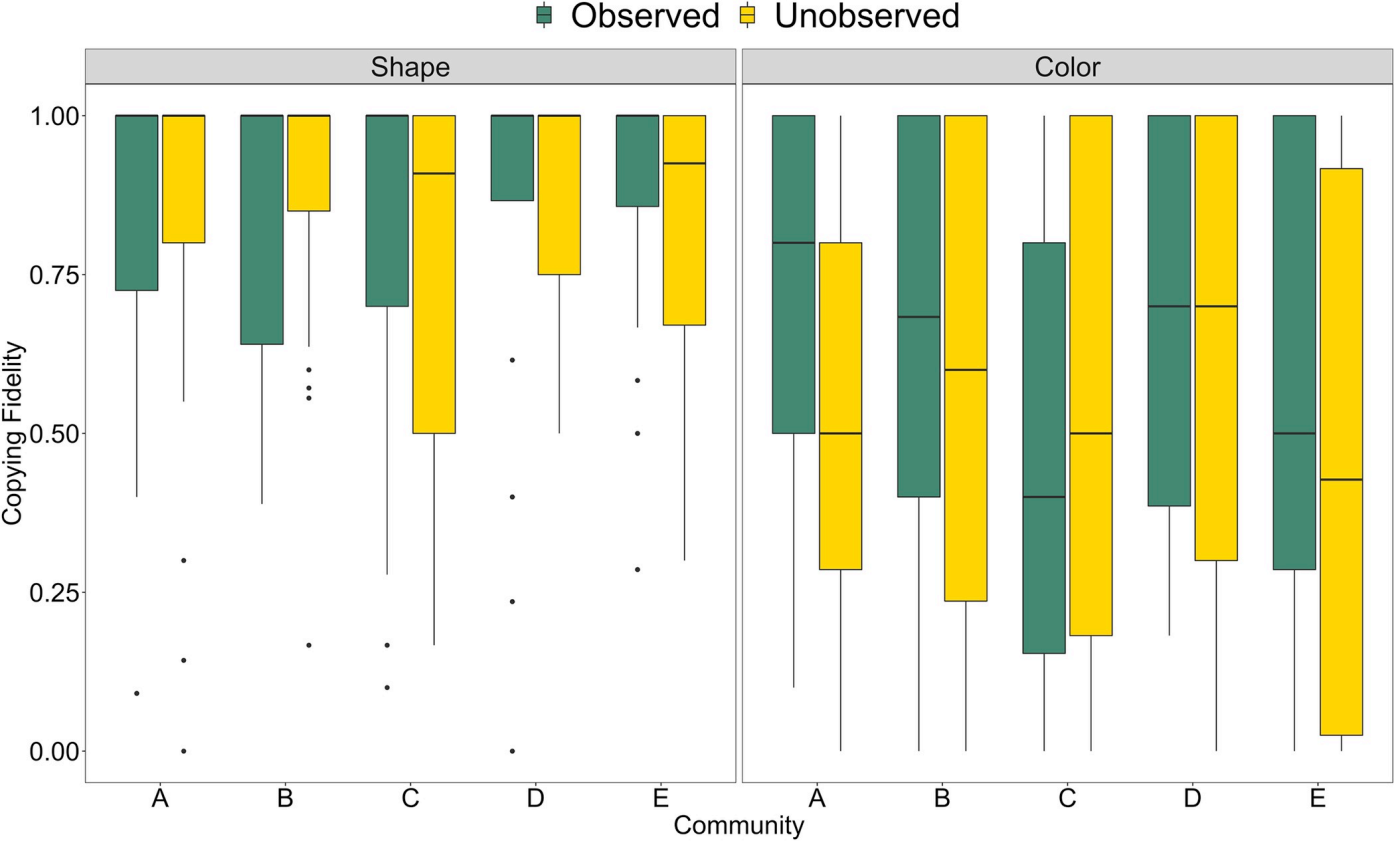
Individual copying fidelity for each community. Note. Individual copying fidelity of the shape (left) and color (right) feature in the observed (green) and unobserved (yellow) condition in each community. The boxes represent the interquartile range (IQR), the bold, horizontal lines within the boxes are medians, the upper vertical lines attached to the boxes extend from the hinge to the largest value no further than 1.5 * IQR from the hinge, the lower vertical lines attached to the boxes extend from the hinge to the smallest value at most 1.5 * IQR of the hinge.

Fig 3 shows individual copying fidelity for each community. Copying fidelity for shape ranges between communities from 0.78 (community C) to 0.87 (community E) and for color from 0.50 (community C) to 0.64 (community A). Model estimates for the varying effect components do not show robust differences (contrast of community-level variation color vs. shape 89% HPDI [-0.87, 0.45]) [H6a]. Being observed does not impact children’s fidelity when copying shape, but increases fidelity when copying color across communities (most clearly in community A) except community C (see S1 Table in S1 File for all values). Between-community differences seem to be higher for copying color in the observed condition (range 0.46 to 0.73) compared to the unobserved condition (range 0.47 to 0.60), but not for copying shape (observed: 0.80 to 0.91, unobserved: 0.77 to 0.88) [H7a].

**Fig 4:**
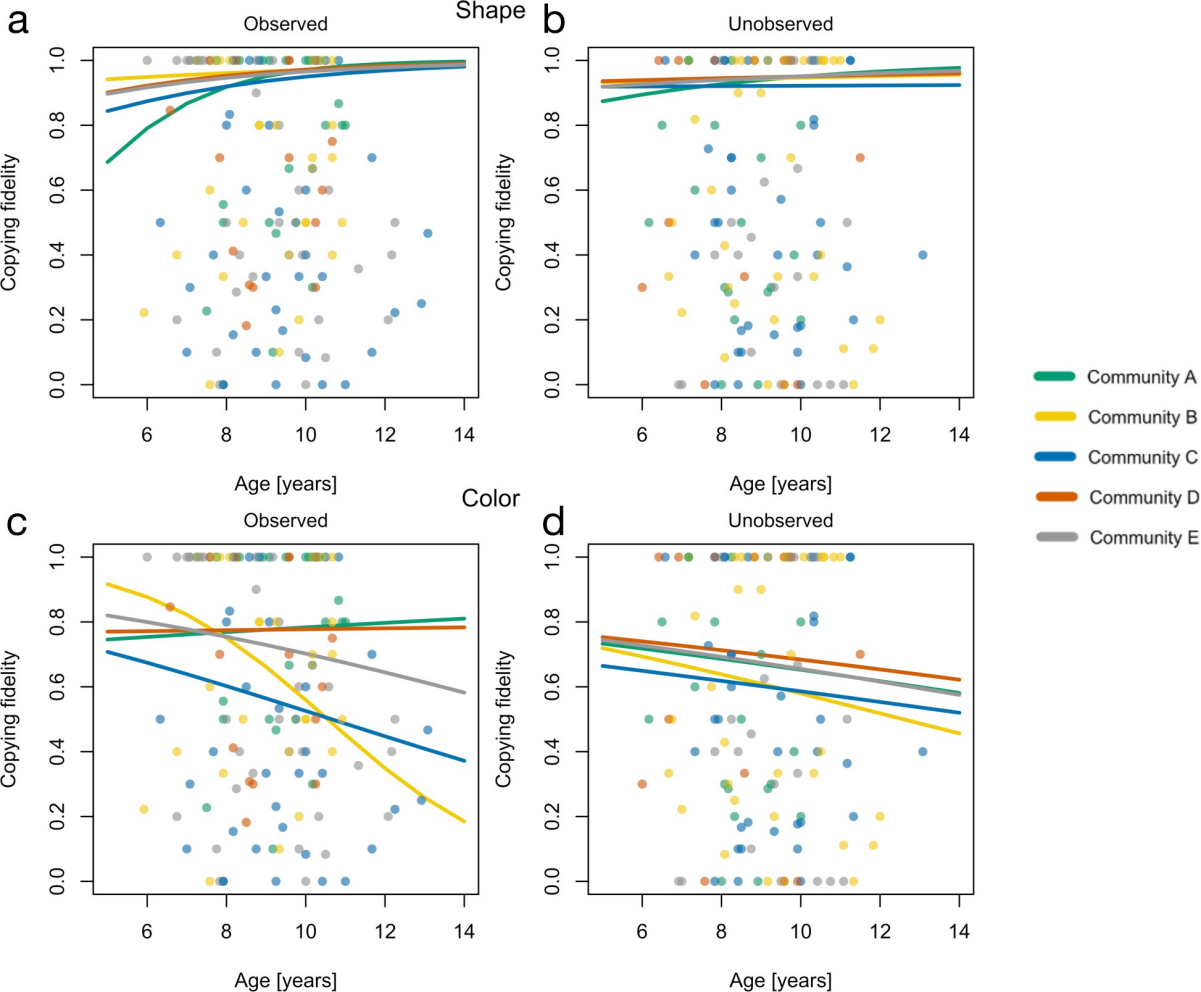
Predicted age-patterns of children’s copying fidelity by feature and condition. Note. Predicted age patterns of children’s copying fidelity by feature and condition based on the Bayesian multilevel model with partial pooling of age. Panels a and b show the age patterns for copying shape, panels c and d show the age patterns for copying colors. Results for the observed condition are shown on the left; results for the unobserved condition are shown on the right.

There is a small trend for children’s copying fidelity to increase with age with respect to shape (overall effect: *β* = 0.14, 89% PI (-0.42; 0.7), see S1 Table in S1 File for all values) but surprisingly a very minor decrease with respect to color (overall effect: *F062* = -0.09, 89% PI (-0.61; 0.43)) [H4a, H4b]. We do not find an increased observation effect in older children (shape: *β* = 0.19, 89% PI (-0.4; 0.77); color: *β* = -0.1, 89% PI (-0.67; 0.47)) [H5]. As depicted in Fig 4, while age patterns are remarkably similar across communities in most conditions (Fig 4A, 4B and 4D), there is substantial variation in age trajectories for copying fidelity of color in the observed condition (Fig 4C) [H6b, H7b].

Exploratorily, we find that children in later experimental positions copy shape with higher fidelity than children in earlier positions (*β* = 0.36, 89% PI (0.18; 0.56)). The same is true for color (*β* = 0.23, 89% PI (0.05; 0.4)), with the exception of a decrease for children in position 6 (see S1 Table for all values and S4 Fig for an illustration in S1 File).

### Transmission chain analysis

**Fig 5:**
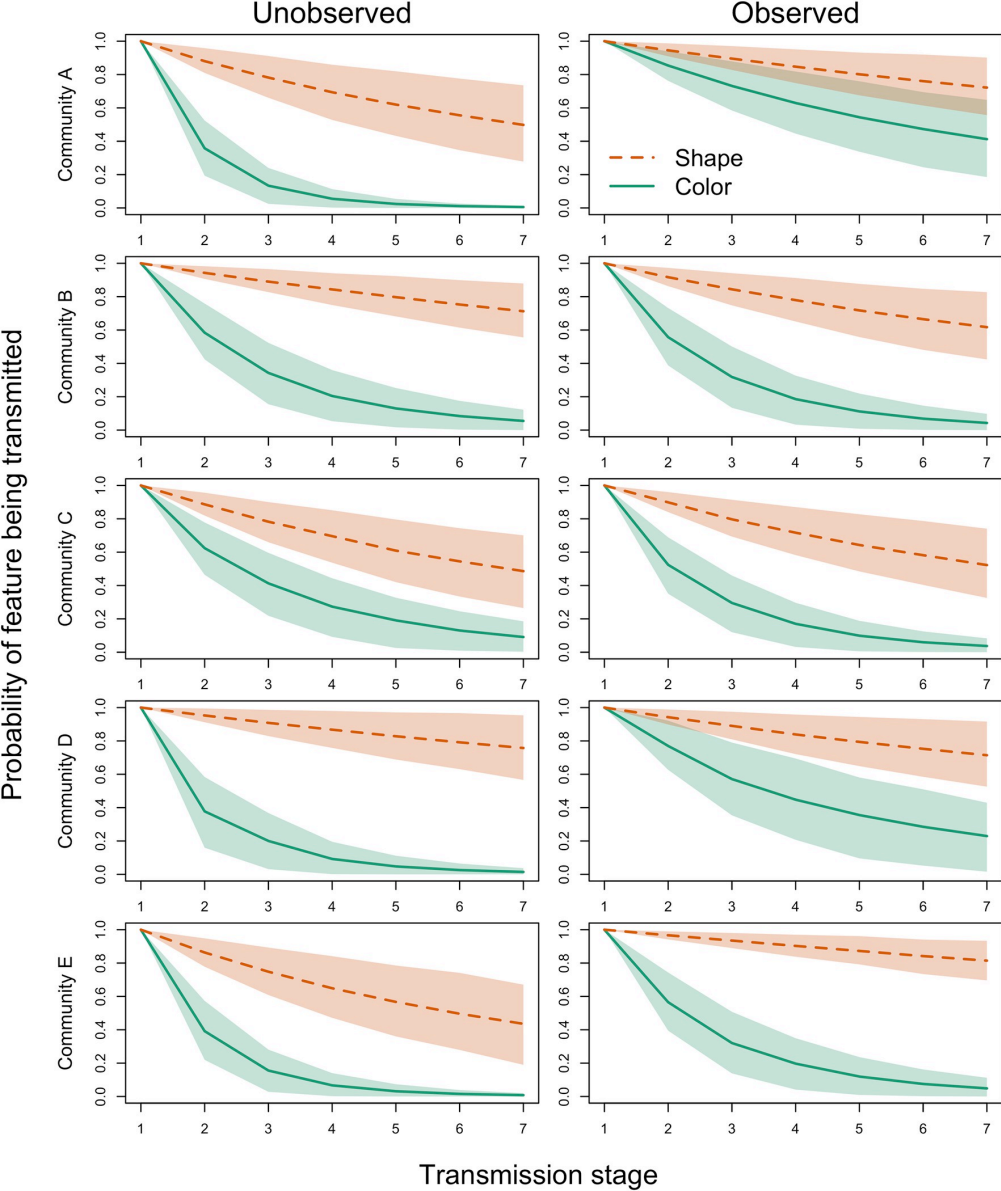
Information flow through transmission chains across five different communities. Note. Mean predicted probabilities (including 89% highest posterior density intervals) for shape (orange) and color (green) features to be transmitted along the stages of the transmission chain in 5 different communities. Results for the observed condition are shown on the left; results for the unobserved condition are shown on the right. Transmission stage 1 resembles the initial marble run given by the experimenters. Estimates were obtained by multiplying preceding conditional transmission probabilities. Note that children in the observed condition were observed if they were in position 2 or higher as position-1-children had no preceding child that could serve as observer.

Fig 5 shows the probabilities of an original building block being reproduced along the chain, separately for different communities, experimental conditions and feature types (see S5 and S6 Figs in S1 File for the respective descriptive information). As for individual copying fidelity, we find that the probability for a shape feature to be reproduced is higher than for a color feature. The probability that a shape feature is maintained in children’s marble runs only mildly decreases as we move through experimental iterations in all communities. The probability that a color feature is maintained, however, sharply decreases after one or two transmission events in several communities. As such, shape features have at least a 44% chance of being transmitted until the end of the chain, i.e., the seventh transmission stage, across communities and conditions (mean probability: 63%, range: 44% to 81%), while color features are often already lost after the fifth transmission stage (mean probability: 9%, range: 1% to 41%; see S2 Table in S1 File).

We also find differences between communities. In community A, after 3 transmission events, there is only around 10% chance for a color feature to be present in the unobserved condition, but a 60% chance in the observed condition. This means, we find, in line with the community results of individual copying fidelity, an observation effect that mainly affects the color feature. The same applies to community D. Community E, however, shows the opposite pattern, where the transmission probability of shape features is increased in the observed compared to the unobserved condition. Community B and C show no observation effect.

## Discussion

In this study we investigated children’s social learning across middle childhood across different communities in Vanuatu while varying the functionality of copyable items and the context of observational learning. We find that ni-Vanuatu children transmit the functional feature *shape* more faithfully than the non-functional feature *color* both in the dyadic transitions as well as along the transmission chains. Being observed influences children’s copying fidelity of color in most communities but not all. Boys tend to copy slightly more faithfully when observed, while girls show the opposite pattern. As children grow older, they copy shape features more and color features less faithfully. Age trajectories show more variation between communities for copying color compared to shape. Children copy more faithfully the further along they are in the transmission chain.

As expected, ni-Vanuatu children seem to be more inclined to copy the functional feature shape than the non-functional feature color [H1]. To understand children’s responses, one has to consider the saliency of our task and social demands [14, 33]. The task was clearly framed as instrumental [34] both through the instruction and the demonstration of the functionality of the marble run. We propose that this enhanced children’s tendency to copy shape, but not color: The color feature was obviously not causally related to a functioning marble run and previous research has shown children’s selectivity in copying based on causal relevance [35]. Moreover, the task of building a functioning marble run was complex and novel–marble runs of this kind are not common on Efate. In consequence, children may have perceived the task as difficult, leading to an enhanced informational motivation to learn from others by applying a ‘copy-when-uncertain’ strategy [36, 37].

Regarding children’s potential social motivations, on the one hand, our task entailed few implicit demands: there was no shared goal, and at most one peer could see the marble run result of a child, lowering potential group pressure. On the other hand, children shared the experience of the test situation and task. In our setup, it was salient to children that they are part of a chain. We found a trend towards more copying in later positions of the chain. The more children have already participated, the more the task might be perceived as a collaborative endeavor [38]. To further test our claims about children’s social motivations, future studies might explicitly highlight color as something socially shared. Moreover, to test whether increased copying with increasing chain position actually supports our claim of social motivation, future research could manipulate whether or not children knew their chain position.

The effects of individual copying fidelity accumulate over iterations, resulting in differential transmission of features throughout the chain: our study, in line with previous research [3, 11], shows that children are able to maintain information over multiple cultural “generations”. Adding to previous research, we find that especially functional features are faithfully transmitted across several generations while non-functional features often get lost along the way.

Against our expectations, we find that the tendency to copy shape features more faithfully than color features increases with children’s age [H4b]. This speaks against a mere prioritization of functional over non-functional copying (see [39] for evidence on children’s hierarchical goal fulfillment) in adaptation to a child’s capacities. Instead, additionally we suggest that social motivations might decrease with age. This is in line with research showing decreasing public conformity over age [12]. As expected, children copied shape more faithfully as they get older [H4a], indicating their increased abilities to learn from others in a selective and rational way [3, 8, 15].

Averaged over communities, we do not detect any observation effect for either feature. In terms of copy shape, we expected this [H2a], in terms of copy color we did not [H2b]. Further, the effect of being observed does not seem to change with age [H5]. This is in line with studies that found stable tendencies of 3- to 7-year old German [15] and 4- to 11-year-old British children [17], but contrasts findings of increasing over-imitation in 3- to 9-year-old Ovambo and Hai||om children [15] and 3- to 11-year-old U.S. American children [16]. We assume that the reason for the overall low observation effect is the very high fidelity even among unobserved individuals (mean of 83% copying fidelity in shape, and 55% copying fidelity in color). This might be caused by the functional constraints on the outcome, but also a potentially exaggerated importance of high fidelity copying among ni-Vanuatu children [34]. Also, the experimental setting might have impacted children’s comfort in the test situation [40] increasing their inclination to copy. Easier and more naturalistic tasks might provide more variance in the influence of being observed.

Looking at communities separately, we find that some of our study communities show the tendency for higher transmission when observed. We find an observation effect in community A for the color feature and in community E for the shape feature. Hence, our results show variation between communities. An explanation for this variation can only be speculative: Based on data that we collected in the same year from parents of participating children [22, Sibilsky A. A Theme and its Variation: Majority-biased Learning in ni-Vanuatu Children. Unpublished PhD. Leipzig: Leipzig University; 2021. [Unpublished]), community A has a relatively high and community E has a relatively low number of ni-Vanuatu languages represented in the community (see S10 Fig from [12]). Thus, for future research, susceptibility to social pressure in observational learning may be of particular interest in explaining how our findings in contemporary communities are related to past cultural transmission processes.

Further, we find more variation in color than shape copying between communities [H6a], particularly among age patterns [H6b], and this variation is even more pronounced among observed children [H7b]. This indicates potentially higher cultural variability of non-functional than functional features, as has been previously found in research on conformity [12, 41]. Sibilsky (Sibilsky A. A Theme and its Variation: Majority-biased Learning in ni-Vanuatu Children. Unpublished PhD. Leipzig: Leipzig University; 2021. [Unpublished]) reports that the sampled communities differ in the emphasis on manners, solidarity with other communities, or the degree to which people are confronted with people speaking another language, i.e., some kind of deviation from the majority. Thus, local developmental contexts might vary in the weighting of certain norms. Of course, individual factors could also underly community variation. DiYanni and colleagues [16] found that in 3 to 7 year old children, the personality factor ‘social desirability’ explained imitation choices of children, whereas ‘vulnerability to social influence’ was more important among 9- to 10-year-olds. An investigation of these factors, in addition to the presented experimental set up, might shed more light on the sources of variation, both between individuals and between communities.

Lastly, we find a slight tendency for boys to copy more faithfully when observed, in contrast to girls, who show the opposite pattern [H3]. This diverges from research on conformity that found higher social influences among ni-Vanuatu girls than boys [12], indicating that girls might be especially susceptible to the influence of groups, while boys are more susceptible to the influence of single peers. This could reflect stronger social expectations for boys to be independent [42], but also a potentially male-favoring task-demand [43]. Relatedly, British boys were more successful than girls in a diffusion chain study on tool use [3]. It would be worth testing our paradigm using tasks that align with ni-Vanuatu girls’ usual activities and preferred games.

A limitation of our study may be that the observing and the observed child frequently tried to interact with each other by looking, whispering, smiling, raising eyebrows or nodding. We tightened the control of children’s interaction in the observed condition after 26 groups were tested to prevent any major influence on our results (see detailed version of the procedure in S1 Section and S3 Table in S1 File for a comparison of the first 26 groups and the rest). We did not detect a systematic difference between children with (observed condition) and without (unobserved condition) the possibility for interaction, so we do not think our results were significantly impacted by children’s interactions.

In this study, we investigated children’s copying fidelity of end-states, individually and throughout a transmission chain, and we additionally varied the functionality of the feature of interest and the copying context to help infer children’s motivations across middle childhood. We found that children are both inclined to and proficient in transmitting a functional feature across multiple experimental generations. Children in our study focus on imitating causally important features but also copy non-functional features albeit with less rigor. This pattern is more evident in older children. The age patterns of copying a non-functional feature show community-level variation, highlighting the influence of local socialization processes. Our study contributes to the understanding of the motivations behind the observational learning of children–the persons who will form society and shape culture in the future. Our results highlight children’s high propensity and developing abilities for observational learning, which ultimately allow for cultural transmission.

## Supporting information

S1 File (DOCX)
