# Correction: Copying fidelity of functional and non-functional features in ni-Vanuatu children: A transmission chain study

**DOI:** 10.1371/journal.pone.0322073

**Published:** 2025-04-04

**Authors:** The PLOS ONE Staff

## Notice of Republication

This article was republished on January 16, 2025, to correct an error in the article title. The word “fidelity” was misspelled in the article title. The correct title is: Copying fidelity of functional and non-functional features in ni-Vanuatu children: A transmission chain study. The correct citation is: Sibilsky A, Colleran H, Deffner D, Haun DBM (2023) Copying fidelity of functional and non-functional features in ni-Vanuatu children: A transmission chain study. PLoS ONE 18(2): e0274061. https://doi.org/10.1371/journal.pone.0274061

## Supporting information

S1 File Originally published, uncorrected article. (PDF)

S2 File Republished, corrected article. (PDF)

## References

[1] Sibilsky A, Colleran H, Deffner D, Haun DBM. Copying fidelity of functional and non-functional features in ni-Vanuatu children: A transmission chain study. PLoS One. 2023;18(2): e0274061. doi: 10.1371/journal.pone.0274061 36757970 PMC9910636

