# Supplementary material for: Copying fidelity of functional and non-functional features in ni-Vanuatu children: A transmission chain study
Source: PLoS One. 2023 Feb 9;18(2):e0274061. doi: 10.1371/journal.pone.0274061 (PMC9910636; doi:10.1371/journal.pone.0274061)
Supplement: S1 File — (DOCX) [file pone.0274061.s001.docx]

SUPPLEMENTARY INFORMATION

# Copying Fidelity of Functional and Non-functional Features in ni-Vanuatu Children:

# A Transmission Chain Study

## S1. Detailed Version of Design and Study Setup

Instructions to the children were standardized and given in Bislama, a *lingua franca* that is understood by all participating individuals. Instructions were translated from English into Bislama, and back-translated, by two local Bislama teachers. Two trained experimenters were present. Experimenter one was a local woman not from any of the participating communities, who gave the instructions to the child. Experimenter two was either a German master’s student or the first author (both White women), who took care of the video recording, counter balancing, live coding and procedure alignment.

It was determined beforehand whether children were tested in the unobserved condition or in the observed condition. Experimenter two called five (unobserved condition) to six (observed condition) children into the study room, telling them that we would like to play a game. Nothing was revealed about the aim of the experiment itself. The study room was divided into a “waiting area” and a “building area”, separated by a waist-high curtain (see Figure 1a and b). The waiting area was used for warm-ups and relaxation for children not currently building a marble run, while the building area was used to assess children’s copying fidelity, keeping them undisturbed but in close proximity to the other children.

In both conditions (observed and unobserved), the procedure was as follows: The children were welcomed by the two experimenters, who introduced themselves and asked them to sit in a circle in the waiting area. Then the warm-up phase began in which children were asked to sort a scattered pile of building blocks to familiarize them with the study materials. Once finished sorting, children drew lots to determine the order of entering the building area. The first selected child followed experimenter one into the building area, while the other children were given pencils and paper for drawing, supervised by experimenter two.

The child sat down in front of a marble run. For the first child in every group, the experimenter showed them a pre-prepared marble run (“Look, here you can see a marble run.”, see Figure 1c). All other children were shown the marble run from the previous round (“Look, here you can see your friend’s marble run.”). The experimenter then demonstrated the function of the marble run by rolling down one or two marbles (“And that’s how a marble run works. One can let a marble roll down”). This step was implemented to ensure a certain kind of building by specifying its use. Finally, the experimenter presented the child with a set of building blocks (see Figure 1d) and asked them to build their own marble run: “Now it is your turn to build and use a marble run. You can use the building blocks in this area. I am going back where the others are. As soon as you have finished, you ring this bell, and I will come back to you.” We framed the instruction in a way that children neither felt pressured to exactly copy the marble run nor to build a completely different marble run. To ensure some degree of comparability, we stuck an adhesive strip on the floor to indicate children to build a linear marble run.

Experimenter one then waited with the other children and experimenter two in the waiting area. When the child rang the bell or when a maximum of four minutes had passed (based on pilot data, this time length proved suitable), experimenter one returned to the building area and asked the child to show their marble run and how it worked. The child subsequently returned to the waiting area while experimenter one took a picture of the marble run and prepared the building area for the next child. To do this, she either dismantled the marble run of the child’s predecessor (leaving the just-built marble run as a model for the next child) or dismantled the child’s marble run if it consisted of fewer than five or more than eleven bricks to avoid very simple and very complex marble runs (leaving the previously built marble run as a model for the next child but pretending that the current child had built it), and prepared the building blocks for the next child. After re-creating the initial set-up, the experimenter one called the next child from the waiting area and the process was repeated. All the children in the group were tested one after the other.

In the observed condition, starting with the second child, participants were not asked to return to the waiting area after building a marble run, but stayed in the building area to observe the following participant. The previous participant was asked to sit diagonally behind the current participant. Experimenter one emphasized that the observing child should do so quietly, without interacting. Thus, each child (except the very last) served both as the creator of a ‘model’ marble run and the observer for the following child. Once the last child built their marble run, we thanked children and they went back to their classrooms.

During data collection, we made the following adaptations: in the first two groups, we initially called the marble run “mox” in order to create a normative structure. This was based on research showing children imitatively learn to use novel things with artificial names in conventional ways and expect others to do so (e.g., (1)). Subsequently, we refrained from calling the marble run “mox” due to the observed high copying fidelity and the risk of a pronounced ceiling effect. Also, we switched from praising the marble runs in the first two groups, to providing neutral or no feedback. Since we already made this change after two groups, we exclude any influence on our results. After we had tested 26 groups (32.5% of all groups), we tightened the control of children's interaction in the observed condition by having experimenter one focus on this child. As we argue in the discussion, we do not think this change affected our results (see also Table S1 for a comparison of the first 26 groups and the rest). After we had tested 37 groups, we influenced the lot-drawing in a way that children for whom we had data from two previous studies (2,3) were not in the first position, as the data of children in the first position could not be analyzed in terms of copying a peer.

**Fig S1. Age of sampled children for the analysis of individual copying fidelity by community**


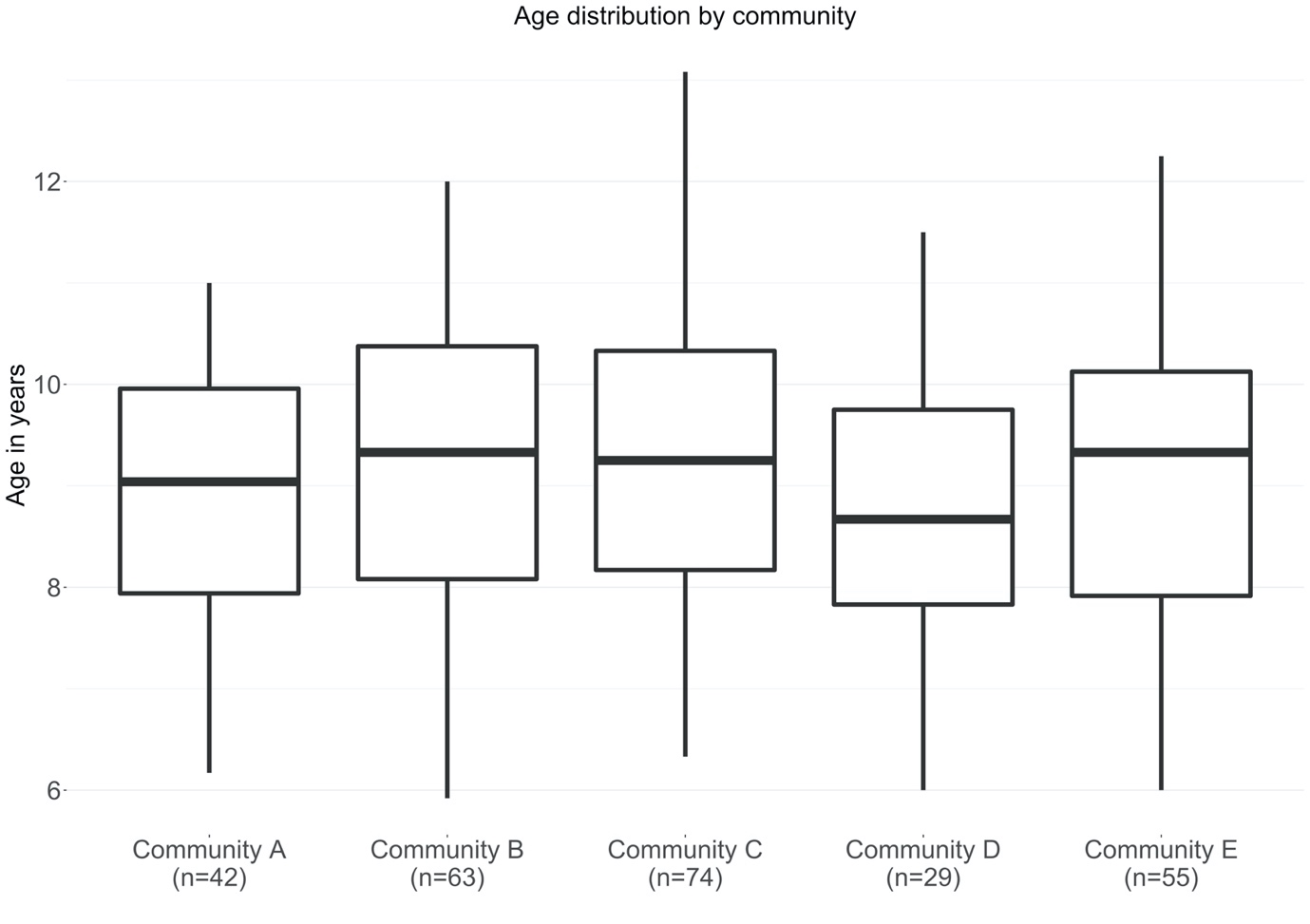


*Note. The boxes represent the interquartile range (IQR), the bold, horizontal lines within the boxes are medians, the upper vertical lines attached to the boxes extend from the hinge to the largest value no further than 1.5 * IQR from the hinge, the lower vertical lines attached to the boxes extend from the hinge to the smallest value at most 1.5 * IQR of the hinge.*

**Fig S2. Age of sampled children for the transmission chain analysis by community**


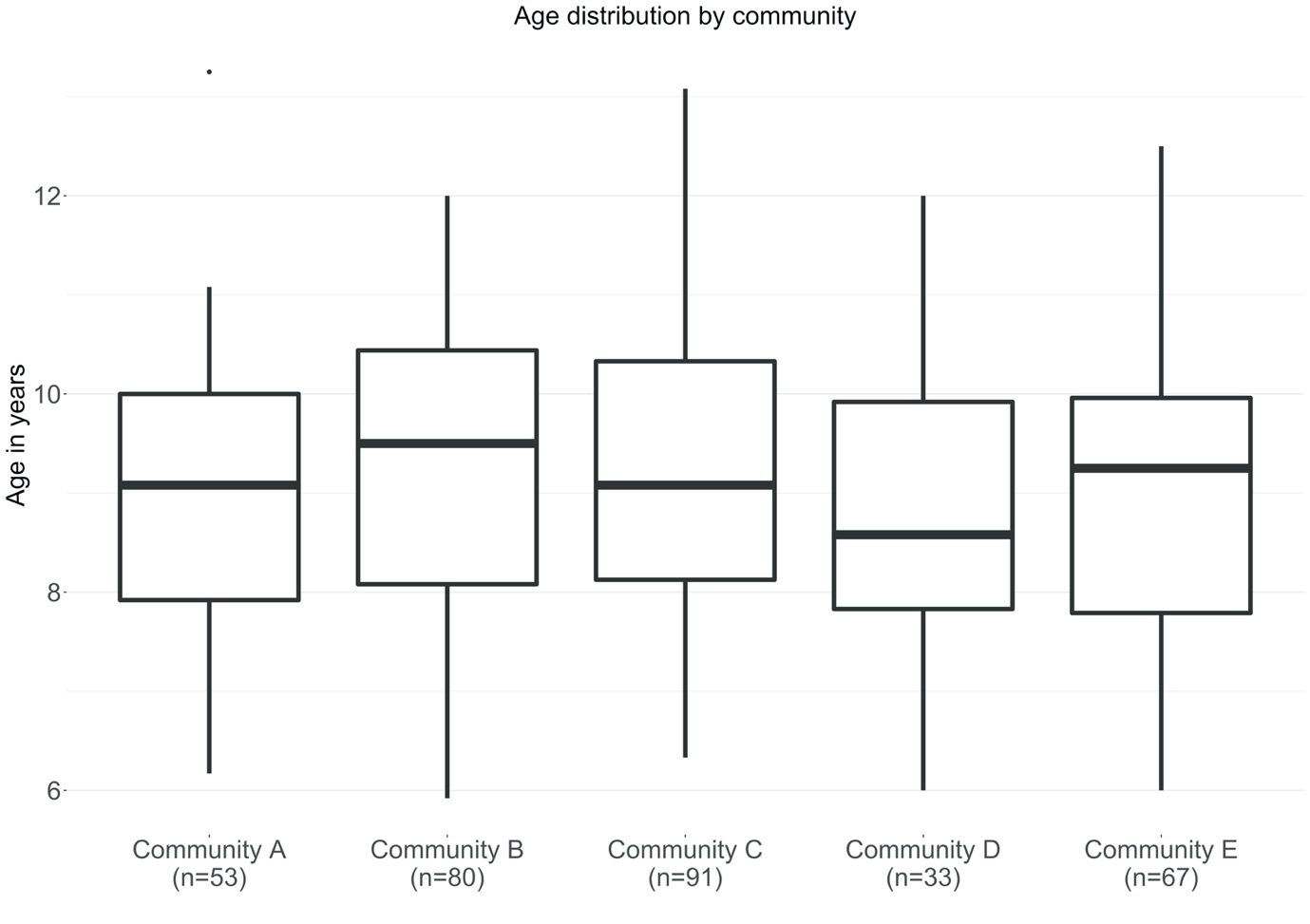


*Note. The boxes represent the interquartile range (IQR), the bold, horizontal lines within the boxes are medians, the upper vertical lines attached to the boxes extend from the hinge to the largest value no further than 1.5 * IQR from the hinge, the lower vertical lines attached to the boxes extend from the hinge to the smallest value at most 1.5 * IQR of the hinge.*

**Fig S3. Coding**

*Note. Panel a) shows how the maximum possible number of 13 matching units is determined: The follower’s marble run has more units, and hence is used to count units. Cubes and ramps count as one unit, all other blocks as two. Each unit is marked by an empty white circle. Panel b) shows how the number of 6 matching shape units is determined: imagine that both marble runs are placed atop each other in the best possible way, then the overlapping units that match in shape are counted. Matching unit are marked as filled white circles, non-matching units as white crosses. Panel c) shows how the number of 7 matching color units is determined: imagine that both marble runs are placed atop each other in the best possible way, then the overlapping units that match in color are counted. Matching unit are marked as filled white circles, non-matching units as white crosses.*

**Table S1. Individual copying fidelity: descriptive mean (M), multilevel model estimates (β), model standard deviation (SD), the borders of the model 89% percentile interval (PI) and 89% highest posterior density interval of selected contrasts (distribution of the expected difference between two levels of a predictor, HPDI).**

| **Variables** | **Mean** | **Model estimates** | | | | | **Contrasts** |
| --- | --- | --- | --- | --- | --- | --- | --- |
|  |  | **estimate** | **β** | **SD** | **PI (5.5%)** | **PI (94.5%)** | **HPDI [5.5%, 94.5%]** |
| **feature** |  |  |  |  |  |  |  |
| shape | 0.84 |  |  |  |  |  |  |
| color | 0.57 |  |  |  |  |  |  |
| **sex** |  |  |  |  |  |  |  |
| shape,girls | 0.82 | a [shape,girls] | 1.29 | 0.51 | 0.45 | 2.07 | contrast shape vs. color  [0.28, 2.26] |
| color, girls | 0.57 | a [color, girls] | 1.22 | 0.5 | 0.43 | 2 |  |
| shape,boys | 0.85 | a [shape,boys] | -0.05 | 0.46 | -0.79 | 0.7 | contrast shape vs. color  [0.64, 2.55] |
| color,boys | 0.57 | a [color,boys] | -0.38 | 0.47 | -1.12 | 0.39 |  |
| **condition** |  |  |  |  |  |  |  |
| shape observed | 0.85 | b_condition [shape] | 0.14 | 0.38 | -0.47 | 0.77 | contrast between conditions [-0.44, 0.79] |
| shape unobserved | 0.83 |  |  |  |  |  |  |
| color observed | 0.59 | b_condition [color] | 0.13 | 0.37 | -0.47 | 0.74 | contrast between conditions [-0.44, 0.76] |
| color unobserved | 0.55 |  |  |  |  |  |  |
| **sex * observation** |  |  |  |  |  |  |  |
| shape observed girls | 0.81 | b_condition_sex [shape, girls] | -0.16 | 0.4 | -0.81 | 0.48 | contrast girls vs. boys:  [-1.32, 0.26] |
| shape unobserved girls | 0.83 |  |  |  |  |  |  |
| shape observed boys | 0.88 | b_condition_sex [shape, boys] | 0.29 | 0.39 | -0.33 | 0.92 |  |
| shape unobserved boys | 0.82 |  |  |  |  |  |  |
| color observed girls | 0.55 | b_condition_sex [color, girls] | -0.15 | 0.4 | -0.78 | 0.5 | contrast girls vs. boys:  [-1.22, 0.35] |
| color unobserved girls | 0.59 |  |  |  |  |  |  |
| color observed boys | 0.62 | b_condition_sex [color, boys] | 0.28 | 0.4 | -0.35 | 0.91 |  |
| color unobserved boys | 0.50 |  |  |  |  |  |  |
| **Table S1. continued** |  |  |  |  |  |  |  |
| **Variables** | **Mean** | **Model estimates** |  |  |  |  | **Contrasts** |
|  |  | **estimate** | **β** | **SD** | **PI (5.5%)** | **PI (94.5%)** | **HPDI [5.5%, 94.5%]** |
| **community** |  |  |  |  |  |  |  |
| community A, shape | 0.83 | c [community A, shape] | 0.17 | 0.42 | -0.32 | 0.93 |  |
| community A, color | 0.64 | c [community A, color] | 0.04 | 0.27 | -0.34 | 0.5 |  |
| community B, shape | 0.87 | c [community B, shape] | 0.27 | 0.43 | -0.19 | 1.04 |  |
| community B, color | 0.61 | c [community B, color] | 0.07 | 0.27 | -0.3 | 0.53 |  |
| community C, shape | 0.78 | c [community C, shape] | -0.17 | 0.35 | -0.76 | 0.27 |  |
| community C, color | 0.50 | c [community C, color] | -0.07 | 0.26 | -0.53 | 0.26 |  |
| community D, shape | 0.84 | c [community D, shape] | 0.16 | 0.43 | -0.38 | 0.97 |  |
| community D, color | 0.64 | c [community D, color] | 0.07 | 0.3 | -0.32 | 0.58 |  |
| community E, shape | 0.87 | c [community E, shape] | 0.28 | 0.45 | -0.2 | 1.14 |  |
| community E, color | 0.52 | c [community E, color] | -0.13 | 0.3 | -0.68 | 0.22 |  |
| **community * condition** |  |  |  |  |  |  |  |
| shape observed community A | 0.85 | b_condition_community [shape, community A] | -0.07 | 0.44 | -0.77 | 0.61 |  |
| shape unobserved community A | 0.82 |  |  |  |  |  |  |
| shape observed community B | 0.86 | b_condition_community [shape, community B] | -0.11 | 0.42 | -0.79 | 0.56 |  |
| shape unobserved community B | 0.88 |  |  |  |  |  |  |
| shape observed community C | 0.80 | b_condition_community [shape, community C] | -0.03 | 0.41 | -0.68 | 0.63 | contrast to community A: [0.03, 1.76] |
| shape unobserved community C | 0.77 |  |  |  |  |  |  |
| shape observed community D | 0.82 | b_condition_community [shape, community D] | -0.02 | 0.44 | -0.73 | 0.69 |  |
| shape unobserved community D | 0.87 |  |  |  |  |  |  |
| **Table S1. continued** |  |  |  |  |  |  |  |
| **Variables** | **Mean** | **Model estimates** |  |  |  |  | **Contrasts** |
|  |  | **estimate** | **β** | **SD** | **PI (5.5%)** | **PI (94.5%)** | **HPDI [5.5%, 94.5%]** |
| **community * condition continued** |  |  |  |  |  |  |  |
| shape observed community E | 0.91 | b_condition_community [shape, community E] | 0.38 | 0.42 | -0.29 | 1.04 |  |
| shape unobserved community E | 0.82 |  |  |  |  |  |  |
| color observed community A | 0.73 | b_condition_community [color, community A] | 0.45 | 0.42 | -0.21 | 1.11 |  |
| color unobserved community A | 0.53 |  |  |  |  |  |  |
| color observed community B | 0.64 | b_condition_community [color, community B] | 0.1 | 0.4 | -0.53 | 0.72 |  |
| color unobserved community B | 0.59 |  |  |  |  |  |  |
| color observed community C | 0.46 | b_condition_community [color, community C] | -0.46 | 0.39 | -1.09 | 0.18 |  |
| color unobserved community C | 0.53 |  |  |  |  |  |  |
| color observed community D | 0.66 | b_condition_community [color, community D] | 0.14 | 0.43 | -0.56 | 0.81 |  |
| color unobserved community D | 0.60 |  |  |  |  |  |  |
| color observed community E | 0.55 | b_condition_community [color, community E] | -0.11 | 0.4 | -0.74 | 0.54 |  |
| color unobserved community E | 0.47 |  |  |  |  |  |  |
| **Table S1. continued** |  |  |  |  |  |  |  |
| **Variables** | **Mean** | **Model estimates** |  |  |  |  | **Contrasts** |
|  |  | **estimate** | **β** | **SD** | **PI (5.5%)** | **PI (94.5%)** | **HPDI [5.5%, 94.5%]** |
| **age** |  | b_age[shape] | 0.14 | 0.35 | -0.42 | 0.7 |  |
|  |  | b_age[color] | -0.09 | 0.33 | -0.61 | 0.43 |  |
| **age * community * condition** |  | b_age_community_condition [shape, community A] | 0.29 | 0.44 | -0.42 | 1 |  |
|  |  | b_age_community_condition [shape, community B] | -0.11 | 0.4 | -0.75 | 0.54 |  |
|  |  | b_age_community_condition [shape, community C] | 0.2 | 0.4 | -0.44 | 0.82 |  |
|  |  | b_age_community_condition [shape, community D] | 0.04 | 0.45 | -0.68 | 0.76 |  |
|  |  | b_age_community_condition [shape, community E] | -0.27 | 0.42 | -0.95 | 0.4 |  |
|  |  | b_age_community_condition [color, community A] | 0.23 | 0.44 | -0.48 | 0.94 |  |
|  |  | b_age_community_condition [color, community B] | 0.25 | 0.41 | -0.41 | 0.9 |  |
|  |  | b_age_community_condition [color, community C] | -0.06 | 0.38 | -0.67 | 0.56 |  |
|  |  | b_age_community_condition [color, community D] | -0.02 | 0.44 | -0.73 | 0.69 |  |
|  |  | b_age_community_condition [color, community E] | -0.51 | 0.41 | -1.16 | 0.13 |  |
| **position** |  | b_position[shape] | 0.36 | 0.12 | 0.18 | 0.56 |  |
|  |  | b_position[color] | 0.23 | 0.11 | 0.05 | 0.4 |  |
| **Table S1. continued** |  |  |  |  |  |  |  |
| **Variables** | **Mean** | **Model estimates** |  |  |  |  | **Contrasts** |
|  |  | **estimate** | **β** | **SD** | **PI (5.5%)** | **PI (94.5%)** | **HPDI [5.5%, 94.5%]** |
| **age * community** |  | b_age_community[shape, community A] | 0.38 | 0.39 | -0.23 | 1 |  |
|  |  | b_age_community [shape, community B] | 0.14 | 0.34 | -0.4 | 0.69 |  |
|  |  | b_age_community [shape, community C] | -0.25 | 0.34 | -0.78 | 0.28 |  |
|  |  | b_age_community [shape, community D] | 0.05 | 0.39 | -0.59 | 0.67 |  |
|  |  | b_age_community [shape, community E] | -0.18 | 0.37 | -0.76 | 0.41 |  |
|  |  | b_age_community [color, community A] | -0.08 | 0.39 | -0.7 | 0.54 |  |
|  |  | b_age_community [color, community B] | 0.14 | 0.35 | -0.41 | 0.7 |  |
|  |  | b_age_community [color, community C] | 0.03 | 0.34 | -0.51 | 0.57 |  |
|  |  | b_age_community [color, community D] | -0.09 | 0.38 | -0.7 | 0.52 |  |
|  |  | b_age_community [color, community E] | -0.11 | 0.38 | -0.71 | 0.5 |  |
| **age * condition** |  | b_age_condition [shape, unobserved] | -0.06 | 0.35 | -0.63 | 0.49 |  |
|  |  | b_age_condition [shape, observed] | 0.19 | 0.37 | -0.4 | 0.77 |  |
|  |  | b_age_condition [color, unobserved] | 0.01 | 0.34 | -0.55 | 0.54 |  |
|  |  | b_age_condition [color,observed] | -0.1 | 0.35 | -0.67 | 0.47 |  |

**Fig S4. Influence of position on copying fidelity**


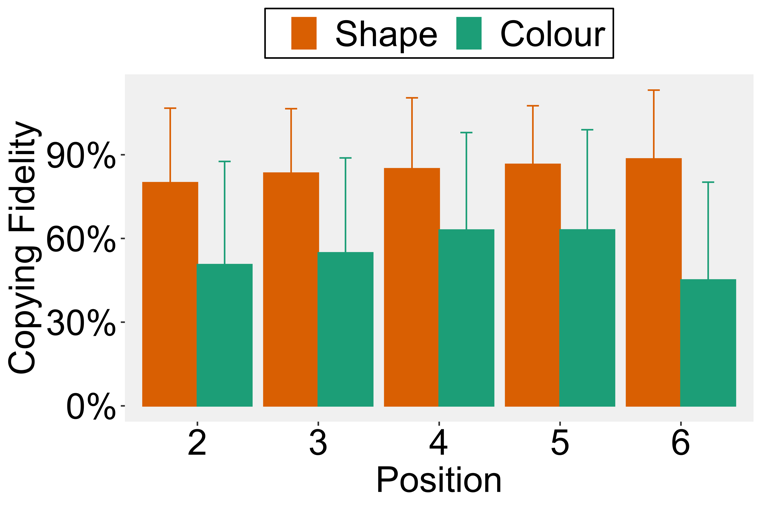


## Fig S5. Descriptive information flow of the color feature through transmission chains across five different communities in the observed and unobserved condition

*
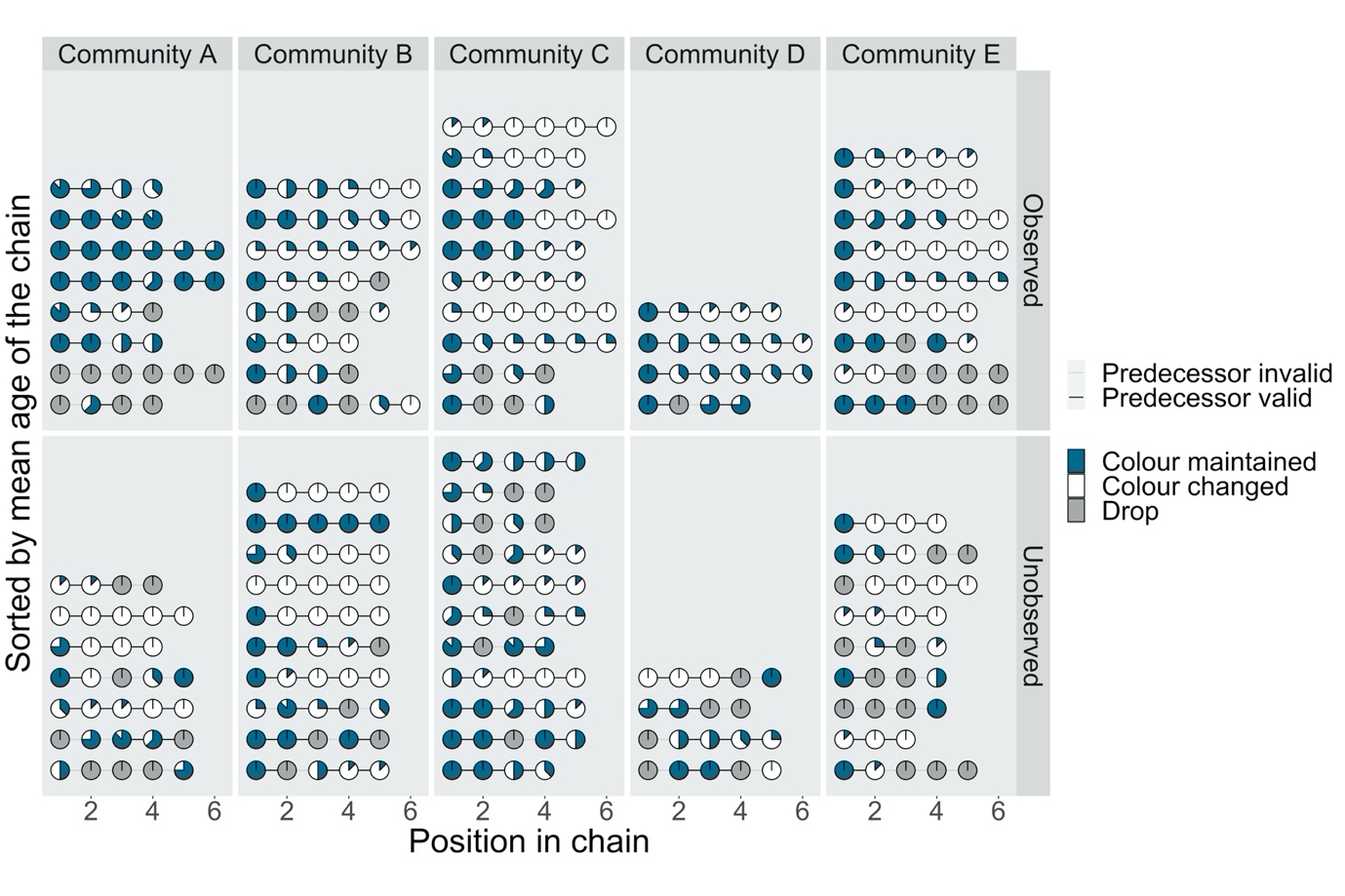
Note. Each circle represents the 8 blocks that a child could have copied from the initial marble run. Circles that are connected by a line belong to one chain. Each eighth of a circle resembles one building block, in blue if the color of the last valid predecessor was maintained, in white if it was not. Grey circles mark drop outs. Black lines connect a circle to a valid predecessor, grey lines indicate an invalid predecessor. In this case the last valid predecessor served as model.*

## Fig S6. Descriptive information flow of the shape feature through transmission chains across five different communities in the observed and unobserved condition


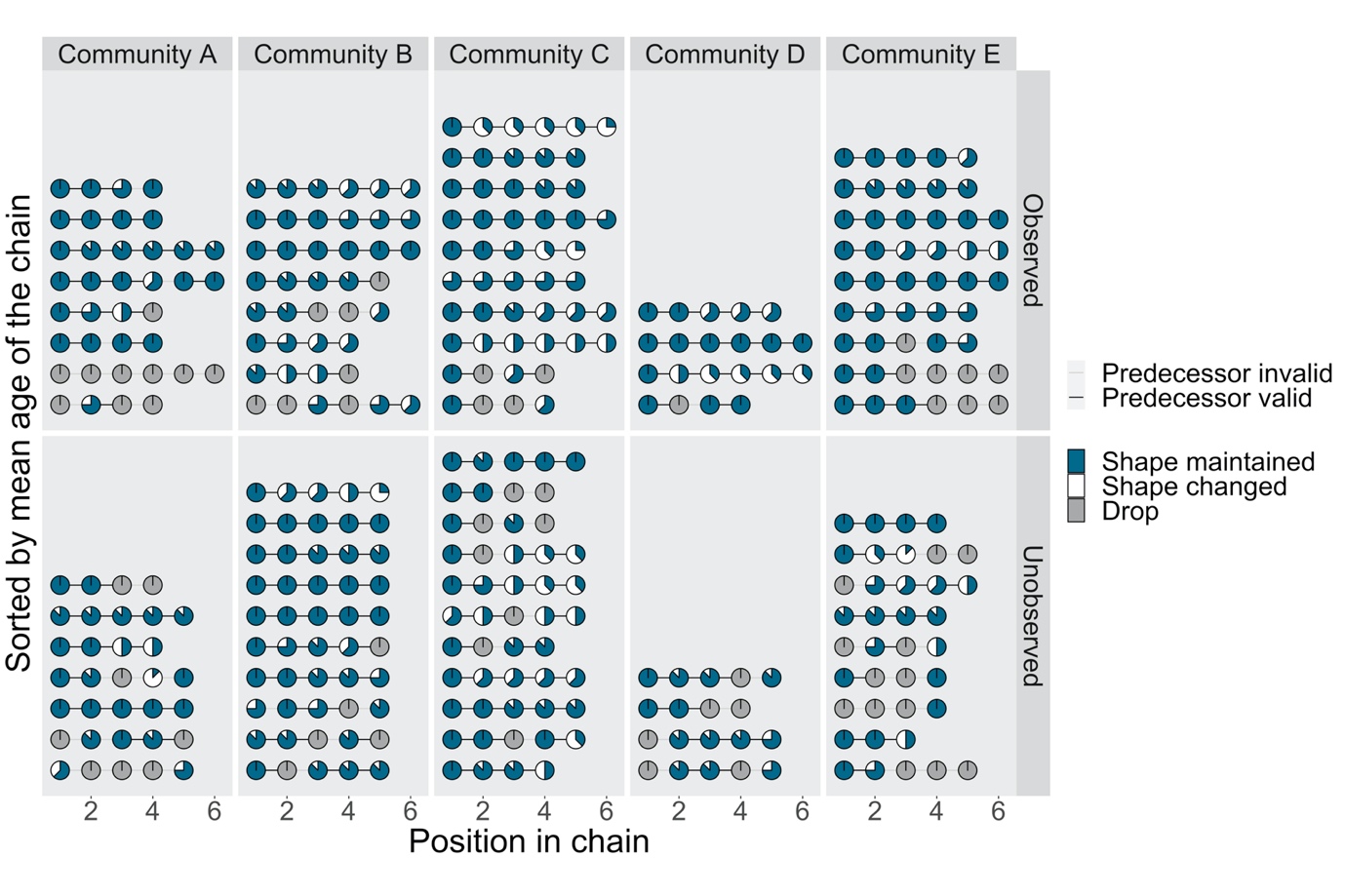
 *Note. Each circle represents the 8 blocks that a child could have copied from the initial marble run. Circles that are connected by a line belong to one chain. Each eighth of a circle resembles one building block, in blue if the shape of the last valid predecessor was maintained, in white if it was not. Grey circles mark drop outs. Black lines connect a circle to a valid predecessor, grey lines indicate an invalid predecessor. In this case the last valid predecessor served as model.*

**Table S2. Transmission chain analysis:** **Probabilities at the 7th transmission stage**

| community | unobserved/color | unobserved/shape | observed/color | observed/shape |
| --- | --- | --- | --- | --- |
| A | 0.005481369 | 0.4980262 | 0.4124771 | 0.7217229 |
| B | 0.05488131 | 0.7125929 | 0.04276139 | 0.6171608 |
| C | 0.09112394 | 0.4861897 | 0.03721866 | 0.5225512 |
| D | 0.01438235 | 0.7575147 | 0.2294861 | 0.7141956 |
| E | 0.007949924 | 0.4356133 | 0.04847082 | 0.8144062 |

**Table S3. Comparison of the first 26 groups and the rest**

|  | **n** | **mean age** | **mean copying fidelity shape** | **mean copying fidelity color** |
| --- | --- | --- | --- | --- |
| **first 26 groups** | 47 (31 boys) | 9.12 years | 89% | 83% |
| **rest** | 89 (48 boys) | 9.25 years | 62% | 57% |

# References

1. Rakoczy H, Warneken F, Tomasello M. The sources of normativity: Young children’s awareness of the normative structure of games. Developmental Psychology. 2008;44(3):875–81.

2. Sibilsky A, Colleran H, McElreath R, Haun DBM. Conformity decreases throughout middle childhood among ni-Vanuatu children: An intracultural comparison. Developmental Psychology. 2021 Sep;57(9):1497–509.

3. Sibilsky A, Colleran H, McElreath R, Haun DBM. Expanding the understanding of majority-bias in children’s social learning. under revision;
