# Supplementary material for: Correction: Copying fidelity of functional and non-functional features in ni-Vanuatu children: A transmission chain study
Source: PLoS One. 2025 Apr 4;20(4):e0322073. doi: 10.1371/journal.pone.0322073 (PMC11970652; doi:10.1371/journal.pone.0322073)
Supplement: S2 File — (PDF) [file pone.0322073.s002.pdf]

PLOS ONE

RESEARCH ARTICLE

Copying fideliy of functional and non-functional features in ni-Vanuatu children: A transmission chain study

Anne Sibilsky[1,2], **Heidi Colleran**[3,4], **Dominik Deffner**[4,5,6], **Daniel B. M. Haun**[1,2]*

1 Department of Comparative Cultural Psychology, Max Planck Institute for Evolutionary Anthropology, Leipzig, Germany, **2** Leipzig Research Center for Early Child Development, Leipzig University, Leipzig, Germany, **3** *BirthRites* Independent Max Planck Research Group, Max Planck Institute for Evolutionary Anthropology, Leipzig, Germany, **4** Department of Human Behavior, Ecology and Culture, Max Planck Institute for Evolutionary Anthropology, Leipzig, Germany, **5** Science of Intelligence Excellence Cluster, Technical University Berlin, Berlin, Germany, **6** Center for Adaptive Rationality, Max Planck Institute for Human Development, Berlin, Germany

* haun@eva.mpg.de

Check for updates

OPEN ACCESS

Citation: Sibilsky A, Colleran H, Deffner D, Haun DBM (2023) Copying fideliy of functional and non-functional features in ni-Vanuatu children: A transmission chain study. PLoS ONE 18(2): e0274061. https://doi.org/10.1371/journal.pone.0274061

Editor: Mark Nielsen, University of Queensland, AUSTRALIA

Received: February 28, 2022

Accepted: August 21, 2022

Published: February 9, 2023

Copyright: © 2023 Sibilsky et al. This is an open access article distributed under the terms of the Creative Commons Attribution License, which permits unrestricted use, distribution, and reproduction in any medium, provided the original author and source are credited.

Data Availability Statement: All analysis scripts and data files are available on the Open Science Framework (https://osf.io/btkgn/?view_only=8722535221b8485d971b3c621c02011a).

Funding: This study was partly funded by the internal budget of the Department of Early Child Development and Culture at Leipzig University (https://www.lfe.uni-leipzig.de/en/home/), and partly by the Max-Planck-Society, a non-commercial, publicly financed scientific organization (no grant number, https://www.eva.

Abstract

Observational learning plays a key role in cultural transmission. Previous transmission chain experiments have shown that children are able to maintain information across multiple generations through observational learning. It still remains unclear how the transmission of functional vs. non-functional information and the effect of being observed unfold across age in different communities. Here, we examine children's copying fidelity in observational learning of 5- to 13-year-olds from five different communities in Vanuatu, both individually (n = 263, 144 boys) and throughout a transmission chain of five to six children (n = 324, 178 boys). We additionally varied the functionality of the feature being copied (shape vs. color) and the copying context (observed vs. unobserved). Further, we also study developmental and cultural variation in the interaction of features and conditions. We find that children transmit the functional feature *shape* more faithfully than the non-functional feature *color*, both in the dyadic transitions as well as the transmission chains with an increasing tendency to do so as they get older. The age patterns show greater variation between communities for color than for shape. Overall, we find that being observed shows no uniform effects but influences transmission differently across communities. Our study shows that children are prone to passing on a functional feature across multiple generations of peers. Children copy non-functional features as well, but with lower fidelity. In sum, our results show children's high propensity and developing abilities for observational learning, ultimately allowing for effective cultural transmission.

Introduction

Culture can be defined as a set of behavioral traditions that are transmitted by social learning [1, 2]. Observational learning, one important social learning mechanism, has been ascribed a key role in human cultural transmission [3]. The observational learning of children is of special

mpg.de/index/). The funders had no role in study design, data collection and analysis, decision to publish, or preparation of the manuscript.

Competing interests: The authors have declared that no competing interests exist.

interest: it equips children with the essential skills they need to become fully-fledged members of the society they live in [4, 5]. As such, children have been labelled as "cultural magnets" [3], and childhood is regarded as a period of massive cultural assimilation [6]. Here, we study observational learning across middle childhood in a country with only approximately 272,000 inhabitants but 138 indigenous languages. Assuming language groups approximate cultural groups, Vanuatu's linguistic diversity indicates extensive cultural diversity. We thereby aim to expand the understanding of both childhood learning and human cultural diversity.

Observational learning is "learning by watching the behavior of others" [3]. Previous research has shown that children are able to learn even if they cannot see the actual action but only its result through so-called *end-state copying*: 19-month-old British infants are able to emulate a required object transformation when exposed to the initial and end-states of an object [7] and four- to five-year-old British children can build a taller spaghetti tower after observing a tower built by others [8].

Combining multiple successive events of end-state copying generates a *transmission chain* [9, 10] which allows researchers to simulate the transmission of cultural information across generations (i.e., cultural evolution) under controlled laboratory conditions: participants in each position of the chain are presented with socially generated information of participants in earlier positions of the chain. Thereby, the fidelity of transfer over repeated transmission events can be tracked by rating the similarity of the respective results. Reindl and Tennie [11] asked four- to six-year-old British and German children to build a spaghetti tower as tall as possible while seeing the towers built by the two previous children. While children's towers showed no indication for cumulative culture (i.e., higher towers in later positions), the similarity of towers was higher within than between chains "indicating the presence of chain-specific design traditions" [11]. In addition, Flynn and Whiten [3] found high fidelity in *how* children achieve an end-state in chains of five children each: 3- and 5-year-olds were asked to remove polystyrene beads from a transparent box using one of two techniques. Children mostly followed the approach they witnessed and thereby were more successful than children in a control condition.

In the present study, we investigate children's fidelity to copy end-states in a transmission chain across middle childhood. We asked children to build a marble run from various building blocks and displayed the marble run built by the previous child. To build a marble run, children could use building blocks in different shapes and colors. The functionality of these two features varies: While the degrees of freedom of the shape feature were restricted, given the aim of building a marble run, the color of the bricks had no influence on the functionality. Therefore, we consider shape as a *functional* feature and color as a *non-functional* feature.

In the following we explain and state our hypotheses H1 to H7 (see also Table 3). We hypothesize that shape will be transmitted more faithfully than color due to the varying functionality of the features [H1]. The reason we predict that color features would be transmitted at all is based on prior studies, relating the copying of non-functional features to chidlren's other, non-informational motivations. Copying a non-functional feature might for example be motivated by social reasons (e.g., affiliation, secure group benefits). To test the possible relevance of social motivations in children's copying, we varied the social context of the situation and tested children in an observed and an unobserved condition. We assume that children copy *shape* (functional feature) equally often in an observed and an unobserved condition [H2a] but copy *color* (non-functional feature) more often when they are observed [H2b]. Previously, in a study on conformity [12]), we showed higher sensitivity to social influences among ni-Vanuatu girls than boys. Therefore, we expect a higher observation effect in girls than boys also in the present study [H3].

To better understand the development of copying fidelity and its cultural variability, we further assessed developmental and cultural variation in the interaction of features and conditions: we expect that older children copy shape [H4a] and color [H4b] more faithfully due to their overall increased cognitive abilities. The age effect might further interact with condition: Previous research has shown that specifically the tendency to copy causally unnecessary actions (so-called "over-imitation" [13, 14]) tended to be higher in older than younger children among 3- to 9-year-old Ovambo and Hai||om children [15] and 3- to 11-year-old U.S. American children [16] (but remained stable in German children [15] and 4- to 11-year-old British children [17]). Furthermore, 3- to 9-year-old Ovambo and Hai||om children [15] also show an increased observation effect with age, leading to higher rates of copying non-functional acts under observation with age. Consequently, we expect an increased observation effect in older children in our study [H5].

Psychologists also increasingly recognize the need for more diverse samples that capture the breadth of human conditions and increase the generalizability of findings across populations [18, 19]. To study cultural variation, we conducted our study in five communities belonging to different ethnolinguistic groups of one island in the Republic of Vanuatu, an archipelago in the southwestern Pacific. Studying copying fidelity across communities in a culturally diverse society allows us to examine cultural variation while minimizing the influence of factors such as varied ecology or living conditions that normally affect cross-country comparisons. As we assume non-functional features to be more culturally variable than functional features, we expect more variation across communities in copying color than in copying shape, both in terms of the general extent of copying [H6a] and also its age patterns [H6b]. Previously, we showed that the availability of a child's behavior to peers can trigger more conformist responses [12]. Therefore, we expect that being observed might have the aggregate effect of exaggerating potential between-community differences in the extent [H7a] and age patterns [H7b] of copying.

Lastly, in addition to the analysis of individual-level copying, we also investigated copying fidelity throughout the transmission chain. This allowed us to see how the hypothesized influence of feature functionality and copying context on individual-level copying would scale up and accumulate over multiple generations.

The cultural diversity in Vanuatu not only allows us to study baseline cultural differences in the end-state copying of children, it could also be associated with an exaggerated importance of high fidelity copying. In addition to three official languages (Bislama, French and English), around 138 indigenous languages of the Oceanic subgroup of the Austronesian language family are spoken in Vanuatu [20] by a population of approximately 300,000 inhabitants [21]. According to Francois [22], this linguistic diversity emerged because language fulfills an emblematic function: it anchors communities in social and geographic space. A necessary condition for the rapid diversification in languages that occurred since first occupation about 3,000 years ago [23, 24] could be high fidelity copying as a way to faithfully transmit linguistic and cultural information to the next generation. This again makes Vanuatu particularly well suited for the study of high fidelity copying.

Methods

Ethics

The study protocols and their application in Vanuatu were approved as part of a broader project using non-invasive behavioral experiments and focusing on early child development, carried out at the Leipzig Research Center for Early Child Development in Germany, and reviewed by the Ethics Committee of the Leipzig Research Center for Early Child

Development in Germany (reference number 169/17-ek). In Vanuatu, permission to carry out the research was given by the Cultural Council of the Vanuatu Cultural Center which regulates all research in the country.

In all participating communities, we obtained informed written consent from the principal of the school in which the experiments were carried out, following extensive discussion of the study protocols. We additionally obtained written consent from the parents of participating children for the collection of experimental data (i.e., study participation, anonymous video recording, and the further usage of the data for scientific purposes) from their children.

Field site

We conducted our study on the principal island of Efate, which has a population of approximately 92,000 inhabitants [21]. Efate belongs to Shefa province and is also home to Vanuatu's capital Port Vila. There are at least three indigenous languages on Efate, Ifira-Mele, Nakanamanga and Nafsan [25], but many other indigenous languages of Vanuatu are now spoken in a wide variety of communities due to in-migration from all over the country (Sibilsky A. A Theme and its Variation: Majority-biased Learning in ni-Vanuatu Children. Unpublished PhD. Leipzig: Leipzig University; 2021. [Unpublished]).

Data were collected in five different schools in distinct communities. According to the residents, these communities each speak a different language. Three of them are rural communities with several hundred residents, located between 25 to 55 km from the capital and accessible in 1 to 1.5 hours by public transportation. The other two study sites have a few thousand inhabitants and are located 7 to 10 km from the capital, which can be reached in 20 to 30 minutes by public transportation. Most of our participants live in brick houses (61%), corrugated-iron huts (22%) or houses made of local materials (15%) (data from interviews with 113 parents of participating children, from (Sibilsky A. A Theme and its Variation: Majority-biased Learning in ni-Vanuatu Children. Unpublished PhD. Leipzig: Leipzig University; 2021. [Unpublished]). Almost all have electricity (96%) either through solar energy (72%) or power lines (27%). Most adults own smartphones (73%), and some have computers (29%) or TVs (22%). Most people have formal education up to primary (43%) or secondary school (50%), but not beyond. About one-third of people are employed (22%) or self-employed (10%). Almost all people have a garden (89%) and use it for their subsistence (95%). The majority of ni-Vanuatu practice slash-and-burn horticulture, and grow staple crops such as yam, taro, banana and manioc (cassava), as well as a variety of vegetables. In addition, they regularly fish (65%), hunt (20%), raise livestock (54%) and buy market-based products. Nearly half of the people reported experiencing economic hardship in the past year (42%) (for more information on all communities, see (Sibilsky A. A Theme and its Variation: Majority-biased Learning in ni-Vanuatu Children. Unpublished PhD. Leipzig: Leipzig University; 2021. [Unpublished]) and Supplementary Methods 1 in [26]).

Sample recruitment and selection

Data were collected between July and September 2018. Participants were recruited out of children from class 1 to 4 who had parental consent. Priority was given to children for whom data already existed, either from our previous study on conformity in 2017 [12] and/or interviews with parents of participating children (Sibilsky A. A Theme and its Variation: Majority-biased Learning in ni-Vanuatu Children. Unpublished PhD. Leipzig: Leipzig University; 2021. [Unpublished]). Usually, we gave consent forms to all children of classes 1 to 4 and conducted the study with almost all for whom we had approval. There were no other criteria, selection

was opportunistic. Where possible, same-sex children were recruited out of the same classroom.

Design and study setup

Instructions to the children were standardized and given in Bislama, a *lingua franca* that is understood by all participating individuals (see S1 Section in S1 File for a more detailed version of the procedure). Two trained experimenters were present. Experimenter one was a local woman not from any of the participating communities, who gave the instructions to the child. Experimenter two was either a German master's student or the first author (both White women), who took care of the video recording, counter balancing, live coding and procedure alignment.

Experimenter two called five (unobserved condition) to six (observed condition) children into the study room, telling them that we would like to play a game. The study room was divided into a "waiting area" and a "building area", separated by a waist-high curtain (Fig 1A and 1B). The waiting area was used for warm-ups and relaxation for children not currently

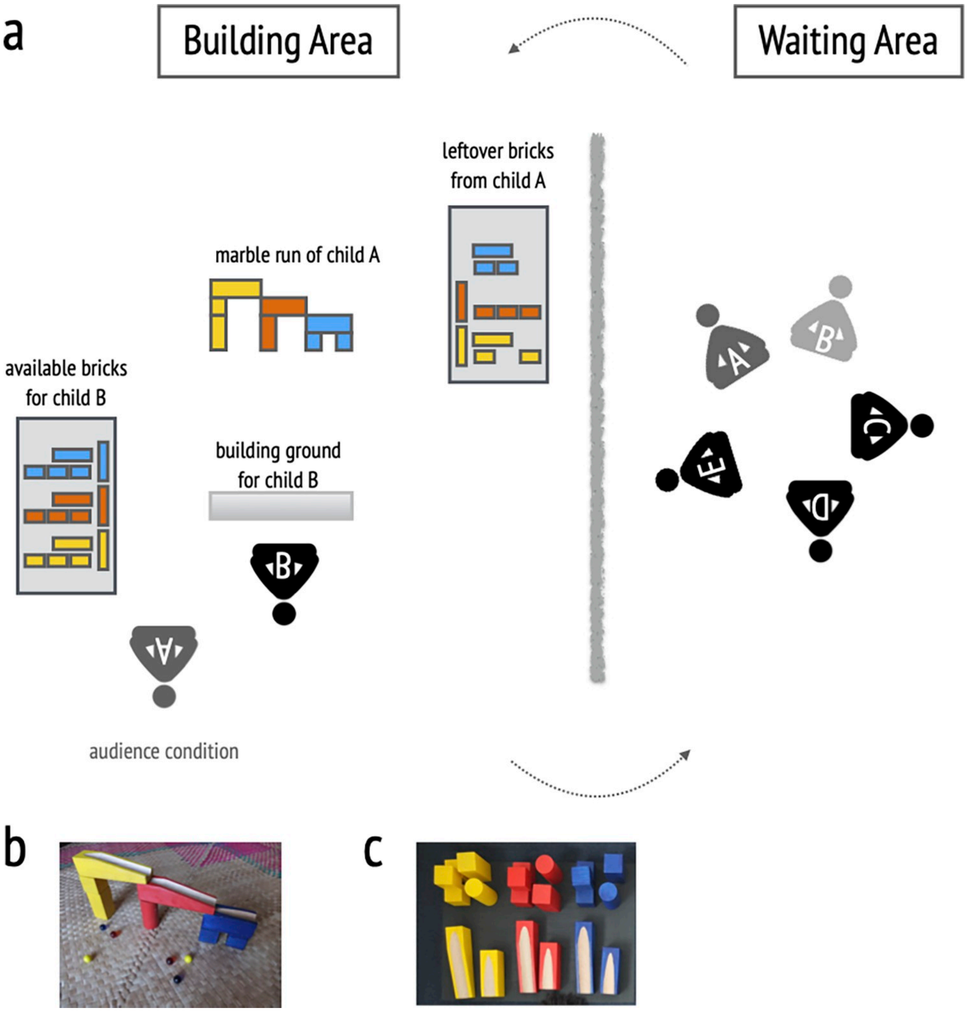

Fig 1. Study design and setup. Note. a) is a schematic illustration of the study setup with the building area on the left and the waiting area on the right which are separated by a curtain. Depending on the condition child A is in sitting in the building area (observed condition) or in the waiting area (unobserved condition) while child B is tested. b) shows the initial marble run given by the experimenter. Panel c) shows the set of building blocks provided to the child.

https://doi.org/10.1371/journal.pone.0274061.g001

building a marble run, while the building area was used to assess children's copying fidelity, keeping them undisturbed but in close proximity to the other children.

In both conditions (observed and unobserved), the procedure was as follows: The children were welcomed by the two experimenters, who introduced themselves and asked them to sit in a circle in the waiting area. In the warm-up phase children were asked to sort a scattered pile of building blocks to familiarize them with the study materials. Once finished sorting, children drew lots to determine the order of entering the building area. The first selected child followed experimenter one into the building area, while the other children were given pencils and paper for drawing, supervised by experimenter two.

The child sat down in front of a marble run. For the first child in every group, the experimenter showed them a pre-prepared marble run (Fig 1C), all other children were shown the marble run from the previous round. The experimenter then demonstrated the function of the marble run by rolling down one or two marbles. This step was implemented to ensure a certain kind of building by specifying its use. Finally, the experimenter presented the child with a set of building blocks and asked them to build their own marble run. We framed the instruction in a way that children neither felt pressured to exactly copy the marble run nor to build a completely different marble run. To ensure some degree of comparability, we stuck an adhesive strip on the floor to indicate children to build a linear marble run.

Experimenter one then went to the waiting area until the child rang a bell or a maximum of four minutes had passed. She then returned to the construction area and asked the child to show their marble run. The child subsequently returned to the waiting area while experimenter one took a picture of the marble run and prepared the building area for the next child. To do this, she either dismantled the marble run of the child's predecessor (leaving the just-built marble run as a model for the next child) or dismantled the child's marble run if it consisted of fewer than five (n = 4) or more than eleven (n = 6) bricks to avoid very simple and very complex marble runs (leaving the previously built marble run as a model for the next child but pretending that the current child had built it), and prepared the building blocks for the next child. We dismantled very simple and very complex marble runs to keep the difficulty of each model run stable within a certain range, thus measuring children's propensity to copy rather than their ability to do so. Then, experimenter one called the next child from the waiting area and the process was repeated.

In the observed condition, starting with the second child, participants were not asked to return to the waiting area after building a marble run, but stayed in the building area to observe the following participant by sitting diagonally behind the current participant. Experimenter one emphasized that the observing child should do so quietly, without interacting. Once the last child built their marble run, we thanked children and they went back to their classrooms.

Participants

80 groups took part in the experiment with a total of 394 children (observed condition: 39 groups, 203 children; unobserved condition: 41 groups, 191 children). Data from 60 children (15% of all children) had to be excluded because they did not build anything but instead looked around or played with the marble (n = 44), they dismantled the marble run that they built themselves or the previous marble run (n = 6), they built something without orientation on the adhesive strip or without a horizontal ramp (n = 6), they used less than five bricks (n = 2), they accidently participated twice (n = 1), or because there was an experimenter error with camera (n = 1).

Table 1. Sample composition for the analysis of individual copying fidelity: Age distribution of children by community and condition for the total sample.

Community		N (male)	mean age	sd age	range age
A		42 (24)	8.9	1.2	6.17,11.00
	observed	22 (15)	9.4	1.1	7.50; 11.00
	unobserved	20 (9)	8.2	1.1	6.17; 10.00
B		63 (32)	9.2	1.4	5.92,12.00
	observed	28 (15)	9.1	1.4	5.92; 10.92
	unobserved	35 (17)	9.3	1.5	6.67; 12.00
C		74 (38)	9.3	1.5	6.33,13.08
	observed	37 (18)	9.4	1.6	6.33; 13.08
	unobserved	37 (20)	9.2	1.4	6.58; 13.08
D		29 (14)	8.8	1.4	6.00,11.50
	observed	16 (7)	9.0	1.2	6.58; 10.67
	unobserved	13 (7)	8.4	1.6	6.00; 11.50
E		55 (36)	9.1	1.5	6.00,12.25
	observed	33 (24)	8.9	1.7	6.00; 12.25
	unobserved	22 (12)	9.3	1.2	6.92; 11.17

https://doi.org/10.1371/journal.pone.0274061.t001

For the analysis of individual copying another 71 children were excluded from the remaining 334 children, because they were in the first position (*age range (years)*: 6.42–13.25; $M = 9.12$; $SD = 1.51$; 37 boys) and thus their data could not be regarded as "copying peers". The final sample includes 263 children (*age range (years)*: 5.92–13.08; $M = 9.12$; $SD = 1.44$; 144 boys) with 136 children in the observed condition and 127 children in the unobserved condition (see Table 1 and S1 Fig in S1 File for the community-wise age distribution).

For the analysis of copying in the transmission chain another 10 children were excluded from the remaining 334 children. These children were successors of children whose marble run had to be dismantled. However, we then accidentally provided the experimenter marble run instead of the marble run of the predecessor. We consider this to be an intervention in the chain of transmission compared to how it would have proceeded uninfluenced and therefore excluded all of the following children. The final sample includes 324 children (*age range (years)*: 5.92–13.25; $M = 9.12$; $SD = 1.46$; 178 boys) with 173 children in the observed condition and 151 children in the unobserved condition (see Table 2 and S2 Fig in S1 File for the community-wise age distribution).

Coding and reliability

We coded the data differently for the analysis of individual copying fidelity and the transmission chain analysis. For individual copying fidelity, the study was coded by experimenter two who rated the copying fidelity of children based on the pictures of the marble runs. The marble runs were divided into coding units, with ramps and cubes being one unit and all other blocks being two units (because two cubes could replace them, see S3 Fig in S1 File for a coding example). These units were then counted in terms of a) the maximum possible number of matching units (i.e., depending on which marble run had more units, the number of units of the previous marble run or the child's marble run), b) the number of matching shape units between the marble run of the child and the previous marble run, and c) the number of matching color units between the marble run of the child and the previous marble run.

85 cases (25% of 334 children) were re-coded by a trained coder blind to the hypotheses. Interrater reliability, calculated with R and the package 'irr' [27], showed substantial agreement (*Cohen's kappa* $_{unit\ maximum}$ = 1, *Cohen's kappa* $_{matching\ shape\ units}$ = 0.729, *Cohen's kappa* $_{matching}$

Table 2. Sample composition for the transmission chain analysis: Age distribution of children by community and condition for the total sample.

Community		N (male)	mean age	sd age	range age
A		53 (31)	8.9	1.4	6.17,13.25
	observed	28 (19)	9.4	1.3	7.42; 13.25
	unobserved	25 (12)	8.4	1.3	6.17; 11.08
B		80 (41)	9.2	1.4	5.92,12.00
	observed	35 (19)	9.2	1.3	5.92; 10.92
	unobserved	45 (22)	9.3	1.4	6.67; 12.00
C		91 (46)	9.3	1.4	6.33,13.08
	observed	48 (22)	9.4	1.6	6.33; 12.92
	unobserved	43 (24)	9.2	1.3	6.58; 13.08
D		33 (16)	8.8	1.5	6.00,12.00
	observed	20 (9)	9.2	1.3	6.58; 12.00
	unobserved	13 (7)	8.3	1.6	6.00; 11.50
E		67 (44)	9.1	1.6	6.00,12.5
	observed	42 (30)	9.0	1.7	6.00; 12.50
	unobserved	25 (14)	9.2	1.5	6.92; 11.83

https://doi.org/10.1371/journal.pone.0274061.t002

$_{color\ units}$ = 0.814, [28]). In cases of disagreement ($n_{unit\ maximum}$ = 0 out of 85, $n_{shape\ matches}$ = 15 out of 85, $n_{color\ matches}$ = 13 out of 85), the rating by experimenter two was preferred due to her greater experience with the coding scheme.

For the transmission of information throughout the chain, we binarised whether the shape and the color of each of the eight building blocks that made up the initial marble run given by the experimenters, which was the same in each group, was reproduced by a child (yes = 1/ no = 0). Once a feature was lost, it could not be rated again in that group: for example, if child A used a red big building block instead of the original yellow big building block, and then child B switched back to a yellow building block, this would still not count as a transmission event.

81 cases (25% of 323 children) were re-coded by a trained coder blind to the hypotheses. Interrater reliability, calculated with R and the package 'irr' [27], showed substantial agreement (*Cohen's kappa = 0.94*, [28]). In cases of disagreement (37 out of 1296), the first author checked the pictures again and decided due to her greater experience with the coding scheme.

Statistical methods

We analyzed all data within a Bayesian modeling framework using the package "rethinking" [29] and "rstan" [30] in R-Studio, R version 4.0.3 [31]. Age was mean-centered.

Individual copying fidelity

For individual copying fidelity, we wanted to know the probability that an individual matches the shape and color features of the previous marble run. We modeled children's copying fidelity using a binomial regression model with a logit link function, separately for shape and color features:

$$y \sim \text{Binomial}(n, p),$$

where copying fidelity y is the number of matching (shape or color) units, n is the maximum possible number of matching units and p is the probability that a child copies a given unit. To investigate which features influence this copying probability, p was defined as a function of the

experimental condition (observed and unobserved), the position of the child in the chain, the community children live in, the child's age, sex, as well as the potential interactions between sex and condition. We also examined the age pattern of copying in the two conditions across communities by evaluating all 2- and 3-way interactions between age, condition, and community. We used a multilevel model to account for the clustered structure of the data, i.e., multiple children belong to the same experimental group and multiple experimental groups live in the same community, and included varying (or "random") effects for each child, group and community (see OSF repository for full model code, [32]). All reported values are based on this model.

Results revealed an interaction between condition, community, and age. To interpret this interaction and better understand varying age trends across communities and conditions, we set up a second model and plotted the model predictions. As we were only interested in this interaction, we did not include other variables (i.e., position and sex). We again used a Bayesian multilevel model to account for the nested structure of the data (children within groups, groups within communities) allowing both the slopes of age and condition as well as the slope of the age-condition interaction to vary by community. This accounts for the fact that different community samples include data on different age ranges.

Transmission chain analysis

In addition to dyadic copying fidelity, we also modeled information flow along the whole transmission chains. For this analysis, we considered for how many iterations of the experiment the building blocks of the original marble run were transmitted, so we ignored the blocks that were introduced at later stages. To compute how far features are expected to travel along the chain, we estimated the transition probabilities for an original feature to be transmitted from the first chain position (i.e., the first iteration of the experiment) to the second, from the second to the third, and so on. This means, conditional on the feature being transmitted up to a certain point, what is the probability that it is also transmitted to the next stage? The outcome variable was whether a given original building block was copied or not by a participant (coded as 1 and 0). We used a Bernoulli likelihood and modeled the six transition probabilities (corresponding to the maximum chain length of six iterations) as ordered-categorical or "monotonic" effects. This means the model assumes that transmission probabilities change in the same direction as we travel along the chain, but the size of the change between, for instance, the first and second stage might differ from the change between the second and third stage. We modeled transmission probabilities separately for the five communities, experimental conditions (observed vs. unobserved) and feature types (shape vs. color) and included individual id and position in the chain as varying effects. The actual transmission stage, which we modeled using monotonic effects, could differ from the position of a child in the chain, because not all children built a marble run that served as a model for the next child in the chain (see *Design and study setup*).

For all analyses, we estimated the posterior probability of parameters conditional on the data using the Hamiltonian Monte Carlo engine *Stan* implemented in Rstan [30] with weakly informative priors to ensure model fit. Inferences are based on 2,000 samples from four chains (after 500 adaptation steps) for a total of 4,000 samples and between 246 to 13462 (individual copying fidelity) and 404 to 6211 (transmission chain) effective samples from the posterior distribution. Model results are reported in detail in S1 Table in S1 File as posterior distributions with mean (β) and 89% percentile interval (PI) on the outcome scale. There we also report the 89% highest posterior density interval (HPDI) of contrasts that we calculated to get the distribution of the expected difference between two levels of a predictor. All analysis scripts and

data files are available on the Open Science Framework (https://osf.io/btkgn/?view_only=8722535221b8485d971b3c621c02011a, [32]).

Results

Individual copying fidelity

Fig 2 shows individual-level copying fidelity. We find that children copy shape more faithfully than color ($M_{Shape} = 0.84$; $M_{Color} = 0.57$; 89% HPDI for contrast [0.23, 0.27]; Fig 2A), which is true for both boys and girls (Fig 2B, see S1 Table in S1 File for all values) [H1, see Table 3 for a summary of all hypotheses]. On average, we do not find that children's copying fidelity (for either shape 89% HPDI [-0.44, 0.79] or color 89% HPDI [-0.44, 0.76]) differed between the observed and unobserved conditions (Fig 2A) [H2a, H2b]. However, boys tended to copy both color (contrast to girls: 89% HPDI [-1.22, 0.35]) and shape (contrast to girls: 89% HPDI [-1.32, 0.26]) features with higher fidelity in the observed compared to the unobserved condition whereas girls show the opposite tendency (Fig 2B) [H3]. Note that distributions for contrasts overlap 0, so our results point to but do not provide conclusive evidence for the observed gender difference.

Fig 3 shows individual copying fidelity for each community. Copying fidelity for shape ranges between communities from 0.78 (community C) to 0.87 (community E) and for color

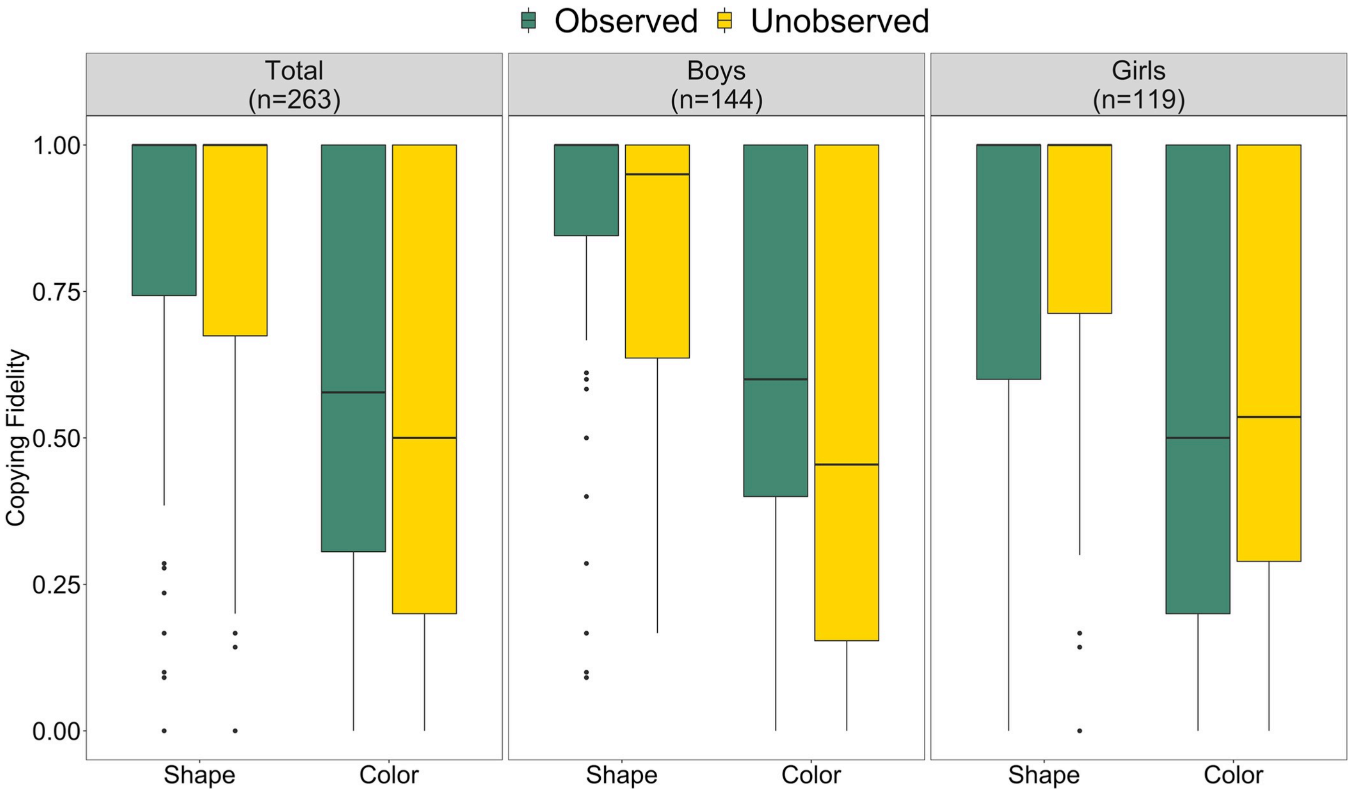

Fig 2. Individual copying fidelity for the total sample, and for boys and girls separately. Note. Individual copying fidelity of the shape and color feature in the observed (green) and unobserved (yellow) condition. a) shows that on average children copy shape more faithfully than color. The boxes represent the interquartile range (IQR), the bold, horizontal lines within the boxes are medians, the upper vertical lines attached to the boxes extend from the hinge to the largest value no further than 1.5 * IQR from the hinge, the lower vertical lines attached to the boxes extend from the hinge to the smallest value at most 1.5 * IQR of the hinge. b) shows that boys tend to copy with slightly higher fidelity when they are observed whereas girls tend to copy with slightly higher fidelity when they are unobserved.

https://doi.org/10.1371/journal.pone.0274061.g002

Table 3. Hypotheses and their results.

Hypotheses		Result
H1	Shape will be transmitted more faithfully than color.	Confirmed
H2a	Children copy *shape* equally often in an observed and an unobserved condition.	Confirmed
H2b	Children copy *color* more often when they are observed.	Not confirmed
H3	We expect a higher observation effect in girls than boys.	Not confirmed
H4a	Older children copy shape more faithfully than younger children.	Confirmed
H4b	Older children copy color more faithfully than younger children.	Not confirmed
H5	Older children show compared to younger children an increased observation effect.	Not confirmed
H6a	There is more variation across communities in copying color than in copying shape in the general extent of copying.	Not confirmed
H6b	There is more variation across communities in copying color than in copying shape in its age patterns.	Confirmed
H7a	Being observed exaggerates potential between-community differences in the extent of copying.	Not confirmed
H7b	Being observed exaggerates potential between-community differences in the age patterns of copying.	Confirmed for color, not confirmed for shape

https://doi.org/10.1371/journal.pone.0274061.t003

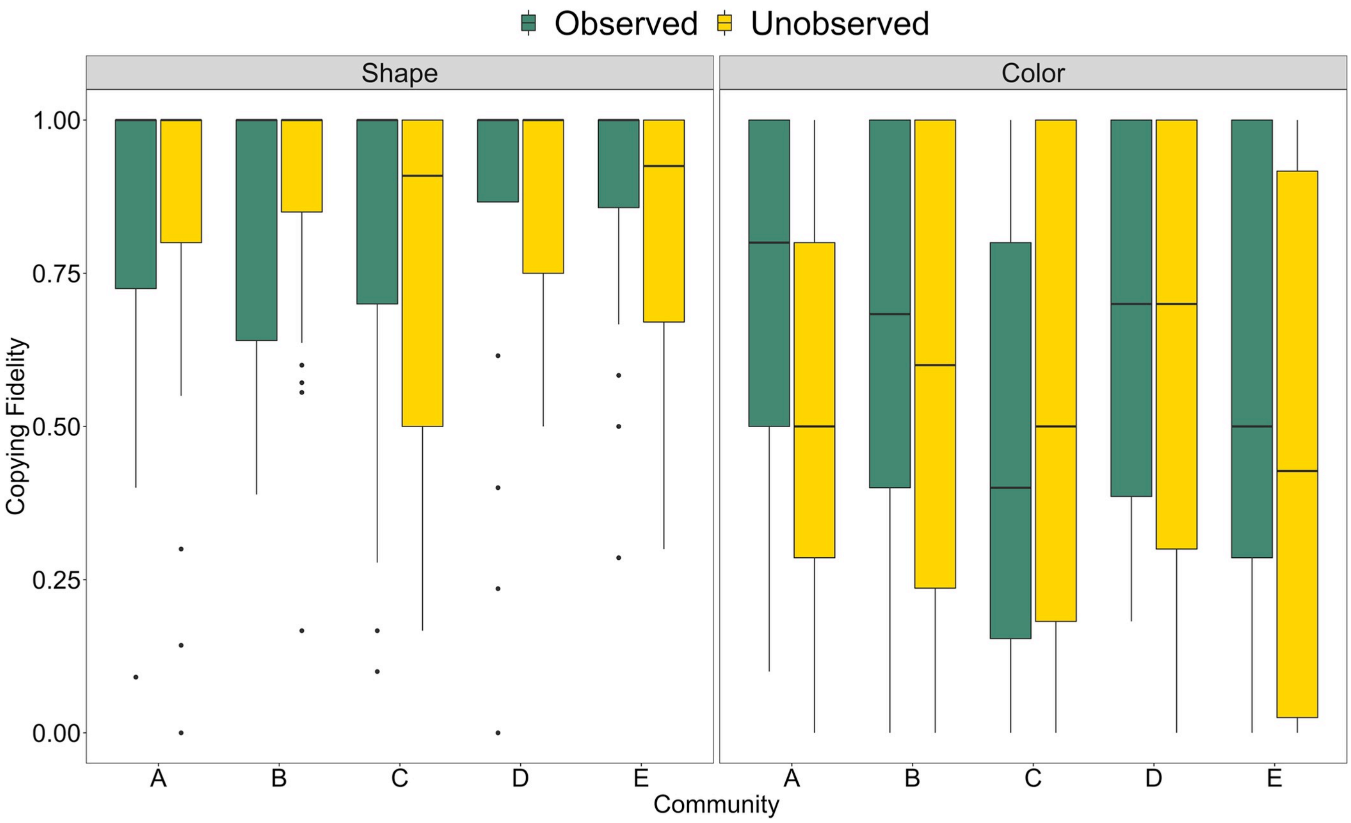

Fig 3. Individual copying fidelity for each community. Note. Individual copying fidelity of the shape (left) and color (right) feature in the observed (green) and unobserved (yellow) condition in each community. The boxes represent the interquartile range (IQR), the bold, horizontal lines within the boxes are medians, the upper vertical lines attached to the boxes extend from the hinge to the largest value no further than 1.5 * IQR from the hinge, the lower vertical lines attached to the boxes extend from the hinge to the smallest value at most 1.5 * IQR of the hinge.

https://doi.org/10.1371/journal.pone.0274061.g003

from 0.50 (community C) to 0.64 (community A). Model estimates for the varying effect components do not show robust differences (contrast of community-level variation color vs. shape 89% HPDI [-0.87, 0.45]) [H6a]. Being observed does not impact children's fidelity when copying shape, but increases fidelity when copying color across communities (most clearly in community A) except community C (see S1 Table in S1 File for all values). Between-community differences seem to be higher for copying color in the observed condition (range 0.46 to 0.73) compared to the unobserved condition (range 0.47 to 0.60), but not for copying shape (observed: 0.80 to 0.91, unobserved: 0.77 to 0.88) [H7a].

There is a small trend for children's copying fidelity to increase with age with respect to shape (overall effect: $\beta = 0.14$, 89% PI (-0.42; 0.7), see S1 Table in S1 File for all values) but surprisingly a very minor decrease with respect to color (overall effect: *F062* = -0.09, 89% PI (-0.61; 0.43)) [H4a, H4b]. We do not find an increased observation effect in older children (shape: $\beta = 0.19$, 89% PI (-0.4; 0.77); color: $\beta = -0.1$, 89% PI (-0.67; 0.47)) [H5]. As depicted in Fig 4, while age patterns are

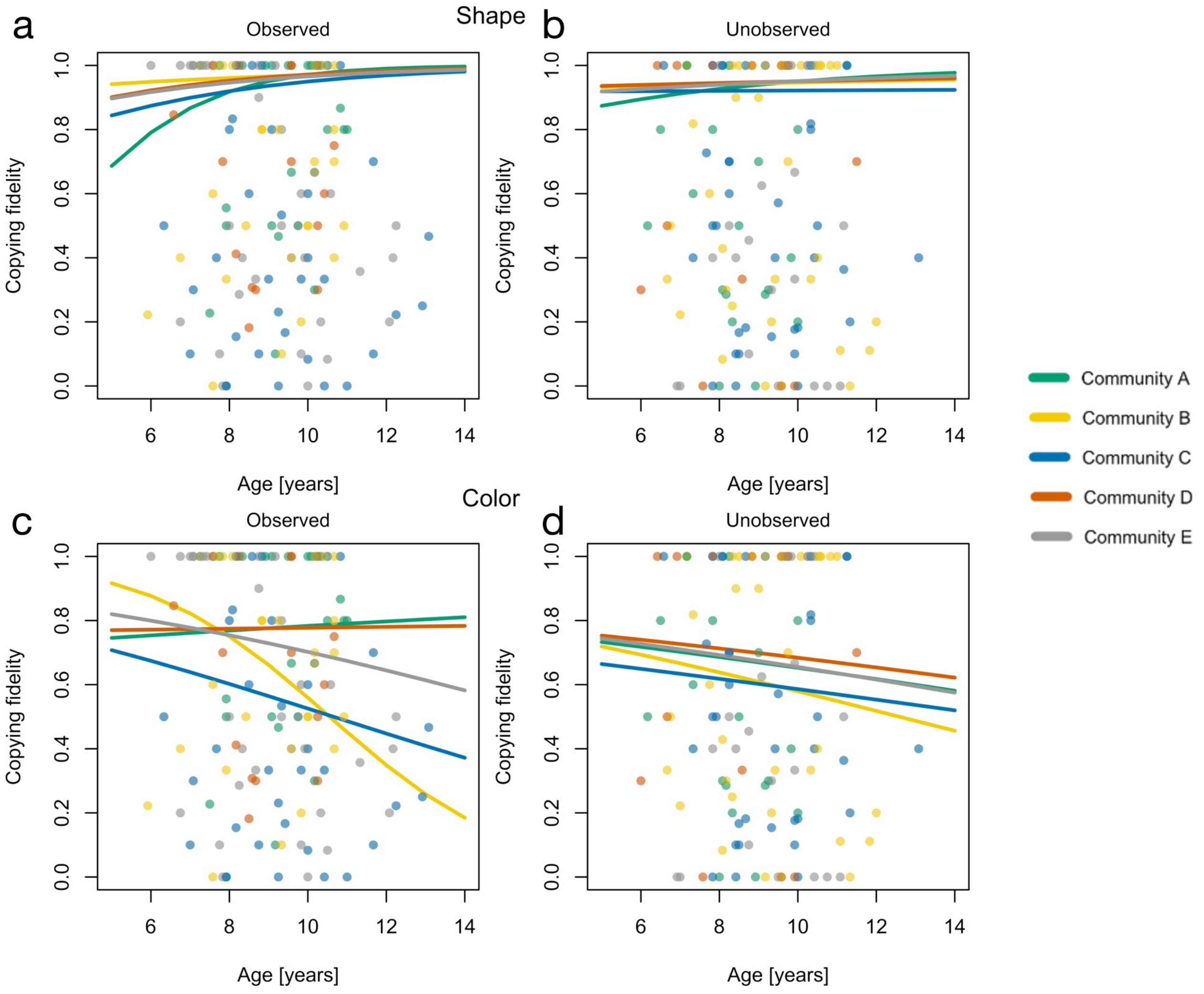

Fig 4. Predicted age-patterns of children's copying fidelity by feature and condition. Note. Predicted age patterns of children's copying fidelity by feature and condition based on the Bayesian multilevel model with partial pooling of age. Panels a and b show the age patterns for copying shape, panels c and d show the age patterns for copying colors. Results for the observed condition are shown on the left; results for the unobserved condition are shown on the right.

https://doi.org/10.1371/journal.pone.0274061.g004

remarkably similar across communities in most conditions (Fig 4A, 4B and 4D), there is substantial variation in age trajectories for copying fidelity of color in the observed condition (Fig 4C) [H6b, H7b].

Exploratorily, we find that children in later experimental positions copy shape with higher fidelity than children in earlier positions ($\beta = 0.36$, 89% PI (0.18; 0.56)). The same is true for color ($\beta = 0.23$, 89% PI (0.05; 0.4)), with the exception of a decrease for children in position 6 (see S1 Table for all values and S4 Fig for an illustration in S1 File).

Transmission chain analysis

Fig 5 shows the probabilities of an original building block being reproduced along the chain, separately for different communities, experimental conditions and feature types (see S5 and S6 Figs in S1 File for the respective descriptive information). As for individual copying fidelity, we find that the probability for a shape feature to be reproduced is higher than for a color feature. The probability that a shape feature is maintained in children's marble runs only mildly decreases as we move through experimental iterations in all communities. The probability that a color feature is maintained, however, sharply decreases after one or two transmission events in several communities. As such, shape features have at least a 44% chance of being transmitted until the end of the chain, i.e., the seventh transmission stage, across communities and conditions (mean probability: 63%, range: 44% to 81%), while color features are often already lost after the fifth transmission stage (mean probability: 9%, range: 1% to 41%; see S2 Table in S1 File).

We also find differences between communities. In community A, after 3 transmission events, there is only around 10% chance for a color feature to be present in the unobserved condition, but a 60% chance in the observed condition. This means, we find, in line with the community results of individual copying fidelity, an observation effect that mainly affects the color feature. The same applies to community D. Community E, however, shows the opposite pattern, where the transmission probability of shape features is increased in the observed compared to the unobserved condition. Community B and C show no observation effect.

Discussion

In this study we investigated children's social learning across middle childhood across different communities in Vanuatu while varying the functionality of copyable items and the context of observational learning. We find that ni-Vanuatu children transmit the functional feature *shape* more faithfully than the non-functional feature *color* both in the dyadic transitions as well as along the transmission chains. Being observed influences children's copying fidelity of color in most communities but not all. Boys tend to copy slightly more faithfully when observed, while girls show the opposite pattern. As children grow older, they copy shape features more and color features less faithfully. Age trajectories show more variation between communities for copying color compared to shape. Children copy more faithfully the further along they are in the transmission chain.

As expected, ni-Vanuatu children seem to be more inclined to copy the functional feature shape than the non-functional feature color [H1]. To understand children's responses, one has to consider the saliency of our task and social demands [14, 33]. The task was clearly framed as instrumental [34] both through the instruction and the demonstration of the functionality of the marble run. We propose that this enhanced children's tendency to copy shape, but not color: The color feature was obviously not causally related to a functioning marble run and previous research has shown children's selectivity in copying based on causal relevance [35]. Moreover, the task of building a functioning marble run was complex and novel–marble runs

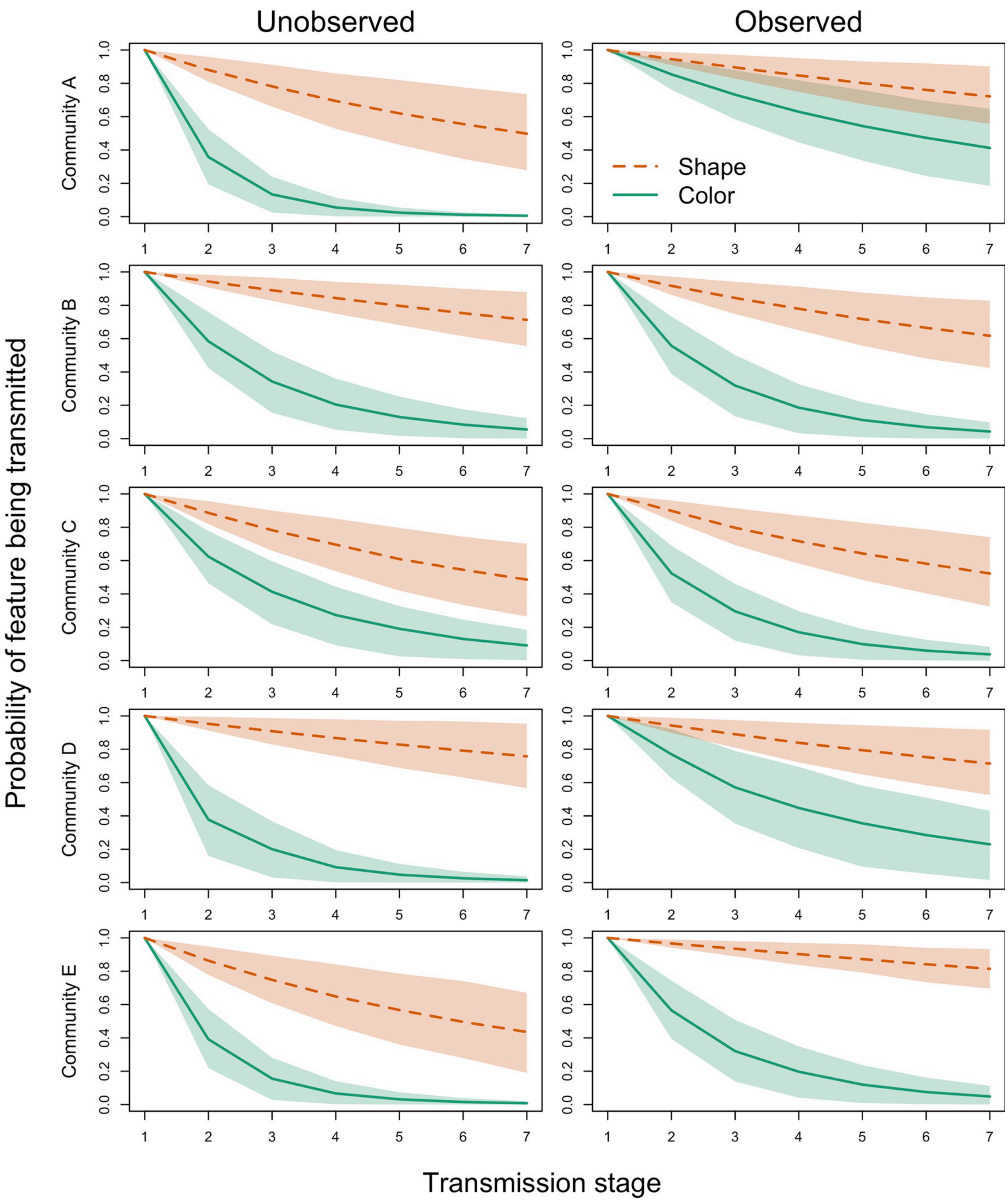

Fig 5. Information flow through transmission chains across five different communities. Note. Mean predicted probabilities (including 89% highest posterior density intervals) for shape (orange) and color (green) features to be transmitted along the stages of the transmission chain in 5 different communities. Results for the observed condition are shown on the left; results for the unobserved condition are shown on the right. Transmission stage 1 resembles the initial marble run given by the experimenters. Estimates were obtained by multiplying preceding conditional transmission probabilities. Note that children in the observed condition were observed if they were in position 2 or higher as position-1-children had no preceding child that could serve as observer.

https://doi.org/10.1371/journal.pone.0274061.g005

of this kind are not common on Efate. In consequence, children may have perceived the task as difficult, leading to an enhanced informational motivation to learn from others by applying a 'copy-when-uncertain' strategy [36, 37].

Regarding children's potential social motivations, on the one hand, our task entailed few implicit demands: there was no shared goal, and at most one peer could see the marble run result of a child, lowering potential group pressure. On the other hand, children shared the experience of the test situation and task. In our setup, it was salient to children that they are part of a chain. We found a trend towards more copying in later positions of the chain. The more children have already participated, the more the task might be perceived as a collaborative endeavor [38]. To further test our claims about children's social motivations, future studies might explicitly highlight color as something socially shared. Moreover, to test whether increased copying with increasing chain position actually supports our claim of social motivation, future research could manipulate whether or not children knew their chain position.

The effects of individual copying fidelity accumulate over iterations, resulting in differential transmission of features throughout the chain: our study, in line with previous research [3, 11], shows that children are able to maintain information over multiple cultural "generations". Adding to previous research, we find that especially functional features are faithfully transmitted across several generations while non-functional features often get lost along the way.

Against our expectations, we find that the tendency to copy shape features more faithfully than color features increases with children's age [H4b]. This speaks against a mere prioritization of functional over non-functional copying (see [39] for evidence on children's hierarchical goal fulfillment) in adaptation to a child's capacities. Instead, additionally we suggest that social motivations might decrease with age. This is in line with research showing decreasing public conformity over age [12]. As expected, children copied shape more faithfully as they get older [H4a], indicating their increased abilities to learn from others in a selective and rational way [3, 8, 15].

Averaged over communities, we do not detect any observation effect for either feature. In terms of copy shape, we expected this [H2a], in terms of copy color we did not [H2b]. Further, the effect of being observed does not seem to change with age [H5]. This is in line with studies that found stable tendencies of 3- to 7-year old German [15] and 4- to 11-year-old British children [17], but contrasts findings of increasing over-imitation in 3- to 9-year-old Ovambo and Hai||om children [15] and 3- to 11-year-old U.S. American children [16]. We assume that the reason for the overall low observation effect is the very high fidelity even among unobserved individuals (mean of 83% copying fidelity in shape, and 55% copying fidelity in color). This might be caused by the functional constraints on the outcome, but also a potentially exaggerated importance of high fidelity copying among ni-Vanuatu children [34]. Also, the experimental setting might have impacted children's comfort in the test situation [40] increasing their inclination to copy. Easier and more naturalistic tasks might provide more variance in the influence of being observed.

Looking at communities separately, we find that some of our study communities show the tendency for higher transmission when observed. We find an observation effect in community A for the color feature and in community E for the shape feature. Hence, our results show variation between communities. An explanation for this variation can only be speculative: Based on data that we collected in the same year from parents of participating children [22, Sibilsky A. A Theme and its Variation: Majority-biased Learning in ni-Vanuatu Children. Unpublished PhD. Leipzig: Leipzig University; 2021. [Unpublished]), community A has a relatively high and community E has a relatively low number of ni-Vanuatu languages represented in the community (see S10 Fig from [12]). Thus, for future research, susceptibility to social pressure in observational learning may be of particular interest in explaining how our findings in contemporary communities are related to past cultural transmission processes.

Further, we find more variation in color than shape copying between communities [H6a], particularly among age patterns [H6b], and this variation is even more pronounced among

observed children [H7b]. This indicates potentially higher cultural variability of non-functional than functional features, as has been previously found in research on conformity [12, 41]. Sibilsky (Sibilsky A. A Theme and its Variation: Majority-biased Learning in ni-Vanuatu Children. Unpublished PhD. Leipzig: Leipzig University; 2021. [Unpublished]) reports that the sampled communities differ in the emphasis on manners, solidarity with other communities, or the degree to which people are confronted with people speaking another language, i.e., some kind of deviation from the majority. Thus, local developmental contexts might vary in the weighting of certain norms. Of course, individual factors could also underly community variation. DiYanni and colleagues [16] found that in 3 to 7 year old children, the personality factor 'social desirability' explained imitation choices of children, whereas 'vulnerability to social influence' was more important among 9- to 10-year-olds. An investigation of these factors, in addition to the presented experimental set up, might shed more light on the sources of variation, both between individuals and between communities.

Lastly, we find a slight tendency for boys to copy more faithfully when observed, in contrast to girls, who show the opposite pattern [H3]. This diverges from research on conformity that found higher social influences among ni-Vanuatu girls than boys [12], indicating that girls might be especially susceptible to the influence of groups, while boys are more susceptible to the influence of single peers. This could reflect stronger social expectations for boys to be independent [42], but also a potentially male-favoring task-demand [43]. Relatedly, British boys were more successful than girls in a diffusion chain study on tool use [3]. It would be worth testing our paradigm using tasks that align with ni-Vanuatu girls' usual activities and preferred games.

A limitation of our study may be that the observing and the observed child frequently tried to interact with each other by looking, whispering, smiling, raising eyebrows or nodding. We tightened the control of children's interaction in the observed condition after 26 groups were tested to prevent any major influence on our results (see detailed version of the procedure in S1 Section and S3 Table in S1 File for a comparison of the first 26 groups and the rest). We did not detect a systematic difference between children with (observed condition) and without (unobserved condition) the possibility for interaction, so we do not think our results were significantly impacted by children's interactions.

In this study, we investigated children's copying fidelity of end-states, individually and throughout a transmission chain, and we additionally varied the functionality of the feature of interest and the copying context to help infer children's motivations across middle childhood. We found that children are both inclined to and proficient in transmitting a functional feature across multiple experimental generations. Children in our study focus on imitating causally important features but also copy non-functional features albeit with less rigor. This pattern is more evident in older children. The age patterns of copying a non-functional feature show community-level variation, highlighting the influence of local socialization processes. Our study contributes to the understanding of the motivations behind the observational learning of children–the persons who will form society and shape culture in the future. Our results highlight children's high propensity and developing abilities for observational learning, which ultimately allow for cultural transmission.

Supporting information

S1 File.
(DOCX)

Acknowledgments

We thank the ni-Vanuatu children for their participation as well as their parents and the principals and teachers of the schools in which we carried out our experiments for their cooperation. We thank the Vanuatu Kaljoral Senta, particularly Directors Asal Lazare and Richard Shing, as well as Evelyn Bulegih, for their support and advice on the design and feasibility of the study. We thank Jule Wolf very much for her help in developing, conducting, and coding the study as well as for research assistance in Vanuatu. We also thank Dorothy Molly and Florina Solomon for research assistance in Vanuatu. We thank Russell Gray for logistical and financial support in Vanuatu. We thank Moritz Schmid and Amelie Conrad for their help in coding. We thank Richard McElreath for additional statistical support, Theo Toppe for the idea of using a marble run and Sarah DeTroy for helpful comments on the manuscript.

Author Contributions

Conceptualization: Anne Sibilsky, Heidi Colleran, Daniel B. M. Haun.

Formal analysis: Anne Sibilsky, Dominik Deffner.

Funding acquisition: Daniel B. M. Haun.

Methodology: Anne Sibilsky, Heidi Colleran, Dominik Deffner, Daniel B. M. Haun.

Supervision: Heidi Colleran, Daniel B. M. Haun.

Visualization: Dominik Deffner.

Writing – original draft: Anne Sibilsky.

Writing – review & editing: Heidi Colleran, Dominik Deffner, Daniel B. M. Haun.

References

1. Boyd R, Richerson PJ. The evolution of ethnic markers. Cultural Anthropology. 1987 Feb; 2(1):65–79.
2. Mace R, Holden C. A phylogenetic approach to cultural evolution. Trends in Ecology & Evolution. 2005 Mar; 20(3):116–21. https://doi.org/10.1016/j.tree.2004.12.002 PMID: 16701354
3. Flynn E, Whiten A. Cultural Transmission of Tool Use in Young Children: A Diffusion Chain Study. Social Development. 2008 Aug; 17(3):699–718.
4. Bjorklund DF, Blasi CH, Ellis BJ. Evolutionary developmental psychology. In: The handbook of evolutionary psychology. 2015. p. 1–21.
5. Geary DC, Bjorklund DF. Evolutionary developmental psychology. Child development. 2000; 71(1):57–65. https://doi.org/10.1111/1467-8624.00118 PMID: 10836558
6. McGuigan N, Burdett E, Burgess V, Dean L, Lucas A, Vale G, et al. Innovation and social transmission in experimental micro-societies: exploring the scope of cumulative culture in young children. Philosophical Transactions of the Royal Society B: Biological Sciences. 2017 Dec 5; 372(1735):20160425. https://doi.org/10.1098/rstb.2016.0425 PMID: 29061897
7. Huang CT, Heyes C, Charman T. Infants' behavioral reenactment of 'failed attempts': Exploring the roles of emulation learning, stimulus enhancement, and understanding of intentions. Developmental Psychology. 2002; 38(5):840–55. https://doi.org/10.1037//0012-1649.38.5.840 PMID: 12220059
8. Reindl E, Apperly IA, Beck SR, Tennie C. Young children copy cumulative technological design in the absence of action information. Sci Rep. 2017 Dec; 7(1):1788. https://doi.org/10.1038/s41598-017-01715-2 PMID: 28496154
9. Caldwell CA, Millen AE. Experimental models for testing hypotheses about cumulative cultural evolution. Evolution and Human Behavior. 2008; 29(3):165–71.
10. Caldwell CA, Millen AE. Social Learning Mechanisms and Cumulative Cultural Evolution: Is Imitation Necessary? Psychol Sci. 2009 Dec; 20(12):1478–83.
11. Reindl E, Tennie C. Young children fail to generate an additive ratchet effect in an open-ended construction task. Smith K, editor. PLoS ONE. 2018 Jun 18; 13(6):e0197828. https://doi.org/10.1371/journal.pone.0197828 PMID: 29912882

12. Sibilsky A, Colleran H, McElreath R, Haun DBM. Conformity decreases throughout middle childhood among ni-Vanuatu children: An intracultural comparison. Developmental Psychology. 2021 Sep; 57 (9):1497–509. https://doi.org/10.1037/dev0001234 PMID: 34929094

13. Hoehl S, Keupp S, Schleihauf H, McGuigan N, Buttelmann D, Whiten A. 'Over-imitation': A review and appraisal of a decade of research. Developmental Review. 2019 Mar; 51:90–108.

14. Lyons DE, Young AG, Keil FC. The hidden structure of overimitation. Proceedings of the National Academy of Sciences. 2007; 104(50):19751–6. https://doi.org/10.1073/pnas.0704452104 PMID: 18056814

15. Stengelin R, Hepach R, Haun DBM. Being observed increases overimitation in three diverse cultures. Developmental Psychology. 2019 Oct 10; 55(12):2630–6. https://doi.org/10.1037/dev0000832 PMID: 31599607

16. DiYanni C, Nini D, Rheel W. Looking good versus doing good: Which factors take precedence when children learn about new tools? Journal of Experimental Child Psychology. 2011 Dec; 110(4):575–91. https://doi.org/10.1016/j.jecp.2011.06.002 PMID: 21782197

17. Marsh LE, Ropar D, Hamilton AF de C. Are you watching me? The role of audience and object novelty in overimitation. Journal of Experimental Child Psychology [Internet]. 2019 Jan [cited 2019 Jan 24]; Available from: https://linkinghub.elsevier.com/retrieve/pii/S0022096518301565 https://doi.org/10.1016/j.jecp.2018.12.010 PMID: 30655097

18. Deffner D, Rohrer JM, McElreath R. A Causal Framework for Cross-Cultural Generalizability [Internet]. PsyArXiv; 2021 Sep [cited 2022 Jan 27]. Available from: https://osf.io/fqukp

19. Nielsen M, Haun D, Kärtner J, Legare CH. The persistent sampling bias in developmental psychology: A call to action. Journal of Experimental Child Psychology. 2017 Oct; 162:31–8. https://doi.org/10.1016/j.jecp.2017.04.017 PMID: 28575664

20. François A, Lacrampe S, Franjieh M, Schnell S, Australian National University, Asia-Pacific Linguistics. The languages of Vanuatu: unity and diversity. 2015.

21. Vanuatu National Statistics Office. National Population and Housing Census [Internet]. 2020. Available from: https://vnso.gov.vu/images/Pictures/Census/2020_census/Census_Volume_1/2020NPHC_Volume_1.pdf

22. François A. The dynamics of linguistic diversity: egalitarian multilingualism and power imbalance among northern Vanuatu languages. International Journal of the Sociology of Language [Internet]. 2012 Jan 22 [cited 2020 Oct 29];2012(214). Available from: https://www.degruyter.com/doi/10.1515/ijsl-2012-0022

23. Lipson M, Skoglund P, Spriggs M, Valentin F, Bedford S, Shing R, et al. Population Turnover in Remote Oceania Shortly after Initial Settlement. Current Biology. 2018 Apr; 28(7):1157–1165.e7. https://doi.org/10.1016/j.cub.2018.02.051 PMID: 29501328

24. Posth C, Nägele K, Colleran H, Valentin F, Bedford S, Kami KW, et al. Language continuity despite population replacement in Remote Oceania. Nat Ecol Evol. 2018 Apr; 2(4):731–40. https://doi.org/10.1038/s41559-018-0498-2 PMID: 29487365

25. Lynch J. South Efate Phonological History. Oceanic Linguistics. 2000; 39(2):320–38.

26. Sibilsky A, Colleran H, McElreath R, Haun DBM. Expanding the understanding of majority-bias in children's social learning. Sci Rep. 2022 Apr 25; 12(1):6723. https://doi.org/10.1038/s41598-022-10576-3 PMID: 35468912

27. Garmer M, Lemon J, Fellows Puspendra Singh I. irr: Various Coefficients of Interrater Reliability and Agreement [Internet]. 2012. Available from: https://CRAN.R-project.org/package=irr

28. Landis JR, Koch GG. The Measurement of Observer Agreement for Categorical Data. Biometrics. 1977; 33(1):159–74. PMID: 843571

29. McElreath R. rethinking: Statistical Rethinking book package. R package version 1. 90; 2019.

30. Stan Development Team. RStan: The R Interface to Stan [Internet]. 2018. Available from: http://mc-stan.org/

31. R Core Team. R: A language and environment for statistical computing [Internet]. Vienna, Austria: R Foundation for Statistical Computing; 2020. Available from: https://www.R-project.org/

32. Sibilsky A, Colleran H, Deffner D, Haun DBM. Copying fidelity of functional and non-functional features in ni-Vanuatu children: A transmission chain study [Internet]. The Open Science Framework; 2022. Available from: https://osf.io/btkgn

33. Over H, Carpenter M. Putting the social into social learning: Explaining both selectivity and fidelity in children's copying behavior. Journal of Comparative Psychology. 2012; 126(2):182–92. https://doi.org/10.1037/a0024555 PMID: 21767011

34. Clegg JM, Legare CH. A Cross-Cultural Comparison of Children's Imitative Flexibility. Developmental Psychology. 2016; 52(9):1435–44. https://doi.org/10.1037/dev0000131 PMID: 27570982

35. Brugger A, Lariviere LA, Mumme DL, Bushnell EW. Doing the Right Thing: Infants' Selection of Actions to Imitate From Observed Event Sequences. Child Development. 2007 May; 78(3):806–24. https://doi.org/10.1111/j.1467-8624.2007.01034.x PMID: 17517006

36. Hamm NH. A Partial Test of a Social Learning Theory of Children's Conformity. Journal of Experimental Child Psychology. 1970; 9:29–42.

37. Morgan TJH, Laland KN, Harris PL. The development of adaptive conformity in young children: effects of uncertainty and consensus. Dev Sci. 2015 Jul; 18(4):511–24. https://doi.org/10.1111/desc.12231 PMID: 25283881

38. Tomasello M, Kruger AC, Ratner HH. Cultural learning. Behav Brain Sci. 1993 Sep; 16(3):495–552.

39. Bekkering H, Wohlschläger A, Gattis M. Imitation of Gestures in Children is Goal-directed. The Quarterly Journal of Experimental Psychology Section A. 2000 Feb; 53(1):153–64. https://doi.org/10.1080/713755872 PMID: 10718068

40. Kline MA, Shamsudheen R, Broesch T. Variation is the universal: making cultural evolution work in developmental psychology. Philosophical Transactions of the Royal Society B: Biological Sciences. 2018 Apr 5; 373(1743):20170059. https://doi.org/10.1098/rstb.2017.0059 PMID: 29440524

41. Corriveau KH, Harris PL. Preschoolers (sometimes) defer to the majority in making simple perceptual judgments. Developmental Psychology. 2010; 46(2):437–45. https://doi.org/10.1037/a0017553 PMID: 20210502

42. Mori K, Ito-Koyama A, Arai M, Hanayama A. Boys, Be Independent! Conformity Development of Japanese Children in the Asch Experiment without Using Confederates. PSYCH. 2014; 05(07):617–23.

43. Cross CP, Brown GR, Morgan TJH, Laland KN. Sex differences in confidence influence patterns of conformity. Br J Psychol. 2017 Nov; 108(4):655–67. https://doi.org/10.1111/bjop.12232 PMID: 27861743
